# Early warning signals for loss of control in complex systems

**DOI:** 10.1073/pnas.2608847123

**Published:** 2026-07-01

**Authors:** Jasper J. van Beers, Marten Scheffer, Prashant Solanki, Ingrid A. van de Leemput, Egbert H. van Nes, Coen C. de Visser

**Affiliations:** ^a^ Department of Aerospace Engineering, Control and Simulation, Delft University of Technology, Delft 2629 HS, The Netherlands; ^b^ Department of Environmental Sciences, Aquatic Ecology and Water Quality Management, Wageningen University and Research, Wageningen 6708 PB, The Netherlands

**Keywords:** tipping points, early warning signals, autonomous systems, robotics, quadrotors

## Abstract

Maintaining stability in feedback systems, from aircraft and autonomous robots to biological and physiological systems, relies on monitoring their behavior and continuously adjusting their inputs. Incremental damage can make such control fragile. This tends to go unnoticed until a small perturbation induces instability (i.e., loss of control). Traditional methods in the field of engineering rely on accurate system models to compute a safe set of operating instructions, which become invalid when the, possibly damaged, system diverges from its model. Here we demonstrate that the approach of such a feedback system toward instability can nonetheless be monitored through dynamical indicators of resilience. This holistic system safety monitor does not rely on a system model and is based on the generic phenomenon of critical slowing down, shown to occur in the climate, biology, and other complex nonlinear systems approaching criticality. Our findings for engineered devices opens up a wide range of applications involving real-time early warning systems as well as an empirical guidance of resilient system design exploration, or “tinkering.” While we demonstrate the validity using drones, the generic nature of the underlying principles suggest that these indicators could apply across a wider class of controlled systems including reactors, aircraft, and self-driving cars.

## Significance

From aircraft to power grids, controlled systems form a crucial part of human societies. Nonetheless, catastrophic failures happen. Many of those arise from the accumulation of incremental problems, such as natural wear and tear, that can go unnoticed until it is too late. We demonstrate that generic indicators of resilience can detect growing instabilities in damaged drones. The generic nature of our approach makes it compatible across diverse controlled systems. This not only allows for on-the-fly warning of instability but also facilitates anomaly detection during manufacturing and promotes proactive maintenance. A complementary application is to use our indicators for exploratory design, allowing one to “tinker” with systems through small adjustments and sensing quickly whether those worsen or improve system resilience.

Controlled systems are deeply intertwined with our daily lives, from power generation and distribution (1, 2) to autonomous vehicles (3–5) but also the human body (6, 7). Across all of these diverse systems, the core objective of the “controller” is the same. That is, to monitor the system’s behavior and guide it to the desired set of states. While this feedback loop typically works well under nominal conditions, any abnormalities or damage incurred by the system can make this feedback relationship fragile (8, 9). In some cases, it leaves the system vulnerable to small perturbations which can induce instability from which it is difficult, or even impossible, for the system to recover in time. We refer to such events as “loss of control.”

Consider, for example, the fatal accident of Sriwijaya Air flight SJ-182 which entered an uncontrolled descent shortly after take-off on January 9, 2021 (10). The subsequent accident report (10) concluded that the buildup of faults within the aircraft, coupled with the overcompensation and delayed reaction of the control systems, ultimately resulted in loss of control. Such loss of stability can likewise occur in systems beyond engineering, such as the decline in postural balance as humans age (11) or even the human body as a whole, wherein cascading failure of several degraded subsystems can lead to a catastrophic system-wide collapse (12). Analogously, no single component caused the fatal accident of Sriwijaya Air SJ-182 alone; instead, it was the complex interconnected system as a whole that failed.

Avoiding such accidents has long motivated innovation in the field of engineering (13, 14). Recognizing that real-world systems are uncertain, the field of robust control pursues the design of controllers which guarantee safety and performance despite model and controller uncertainties (15–17). Similarly, much work is being done to design specialized fault-tolerant controllers that can maintain safety across an assortment of fault scenarios (9, 18, 19). Another popular view on safety is to consider the system’s capabilities through reachability analysis (20, 21). The goal is to establish a region of attraction for a safe set of states to avoid collisions (22–24) or to remain within a prescribed safe operational envelope (25–27). Advancements in these fields have improved safety across many engineered systems. However, these control theoretic approaches all share a key limitation in that they rely on (a collection of) system models to establish safety. When these models become misaligned with reality, even well-engineered systems can suffer loss of control.

An alternative solution is therefore to find ways of anticipating loss of control that do not rely on a preexisting system model. Here we develop such a model-free approach based on methods from dynamical systems theory for which a feedback instability can be considered a critical transition. This allows for its prediction via generic indicators of resilience (28–30). Using drones, we show that a controlled system’s proximity to loss of control can indeed be monitored holistically, without the need for its system model, through the generic phenomenon of critical slowing down (CSD).

CSD has been shown to occur in ecological (31–33), biological (34–36), and many other complex dynamical systems (37–39). This is most commonly done by determining the lag-1 autocorrelation and variance in a moving window along timeseries of the system states. Moreover, it has recently been shown that CSD can be measured in bursts: brief periods of measurement in intervals preceding a critical transition (40). Similarly, we monitor the step-wise deterioration in resilience of a quadrotor (a four propeller helicopter) subject to propeller damage increased in increments up to the point of instability. We show that the quadrotor can tolerate blade damage in some operating conditions but loses control completely in others. Therefore, these flight experiments serve as a proxy for situations where gradual damage can be incurred by a controlled system, nudging it closer to instability, even if it remains deceptively operational. This insidious approach toward instability is reflected by the dynamical indicators of resilience.

## Generic Monitoring of Loss of Control

Many controllers rely on feedback information to guide a system toward the desired behavior. Such a system is “closed-loop” in the sense that the controller relies on state information (measurements) to facilitate control. The characteristics of this feedback information thus affect the resilience of the controller. For instance, our ability to maintain postural balance benefits from visual feedback and any impairments (e.g., closed-eyes) inhibit this ability (11). In engineered systems, the feedback signal is often imperfect, containing sensor dynamics, measurement delays, and inaccuracies (perturbations) such as bias and noise. These imperfections misinform the controller and can thus lead to feedback instability. Illustrative examples of how such instability can arise in controlled systems are given in *SI Appendix*, section S1. In control theory, we refer to this as “closed-loop instability”; the system in combination with the controller is unstable.

The issue of instability becomes especially problematic for controlled systems which not only suffer from measurement imperfections but also endure gradual changes to the system dynamics. Take, for instance, natural degradation due to wear and tear, or component damage itself, or even (software) system upgrades and improvements. The consequences of these changes on stability can go unnoticed as the controller seeks to maintain the controlled equilibrium until an imminent instability suddenly becomes apparent.

There is growing evidence that, across wildly different systems, such feedback instability can be predicted through CSD (29, 30, 36, 41). However, current demonstrations for engineered systems (29, 30) do not consider the influence of a controller that actively drives the system. Due to the persistent feedback influence of the controller, alongside the mechanisms which govern the inherent stability of the system itself, it is often difficult to generically ascertain the exact moment of instability through CSD alone. Thus, rather than predicting this exact moment in the conventional way, we instead apply the generic indicators of resilience to monitor shifts in stability for the current controlled system as a whole. This nuanced difference affords us insights into a deterioration in controlled system resilience without knowing the exact mechanisms that culminate in this nudge toward instability. In fact, the generic indicators of resilience are consistent with traditional system model dependent control theoretic methods for assessing stability, robustness, and resilience (*SI Appendix*, section S3). In summary, also in a controlled system we can expect the characteristic symptoms of CSD as it approaches instability.

### Quadrotors as an Example.

**Fig. 1.**
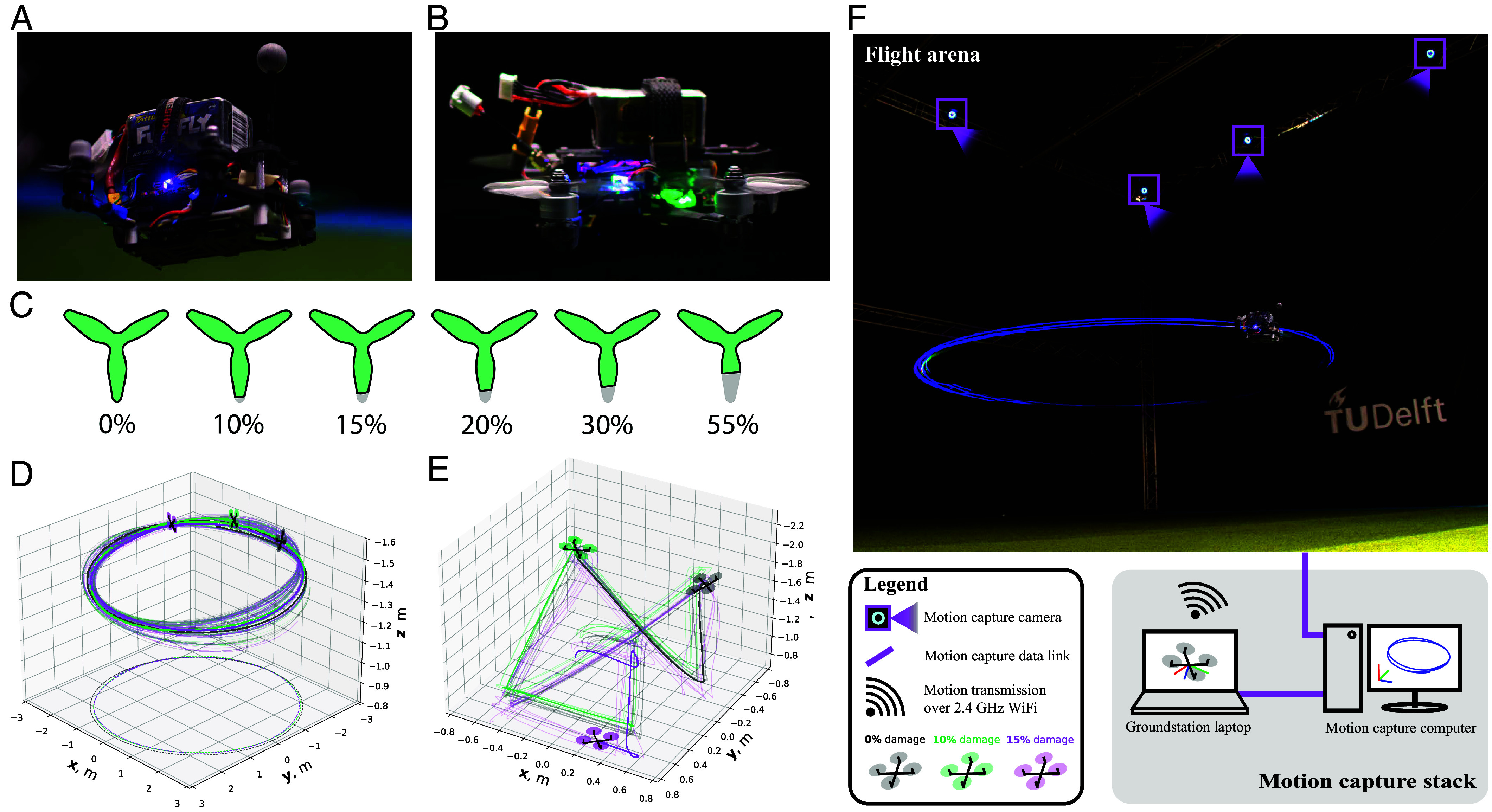
Quadrotor flight experiments with incremental blade damage. We conduct experiments with two quadrotors: the autonomous DragonFly (*A*) controlled using the INDIFlight controller and the human-piloted HoverFly (*B*) operating the Betaflight controller. Flight experiments are conducted with various degrees of blade damage (*C*) from healthy (0% damage) to 55% blade tip damage. We task the DragonFly with autonomous hovering and trajectory tracking tasks (see *D* for forward circular flight and *E* for tracking the vertices of a three-dimensional box in position; the opaque lines indicate independent flights while the solid lines depict a portion of the flight trajectory at various discrete damage levels). Conversely, the nonautonomous HoverFly is only flown in hover. The autonomous flights of the DragonFly are facilitated by a motion capture system (OptiTrack) which observes the quadrotor state and relays these measurements via WiFi on a groundstation laptop (*F*).

We apply these dynamical indicators of resilience to monitor shifts in stability of quadrotors (Fig. 1 *A* and *B*) subject to incremental propeller damage (Fig. 1*C*) up to the point of instability. The quadrotor can be regarded as a good example of a complex feedback system; its dynamics are highly nonlinear and it is subject to many disturbances that are difficult to predict or measure, such as aerodynamic interactions and component manufacturing inconsistencies (42). These challenges are compounded by the underactuated (i.e., fewer inputs than system states) nature of the quadrotor, making it a popular platform upon which to design and evaluate advanced control algorithms (42, 43).

To this end, agile flight is afforded by clever engineering that mitigates many of the challenges associated with controlling the quadrotor. For example, an array of targeted (and often adaptive) feedback filters limit the severity of vibrations and sensor noise generated by the aerodynamics of the propellers and surrounding airflow (44, 45). Such effects would otherwise destabilize the controller (46) through self-reinforcing feedback loops (*SI Appendix*, section S1, Example 2). However, the use of these filters comes at the cost of additional feedback delays which can also degrade system stability (*SI Appendix*, section S1, Example 1).

Clearly, such aggressive engineering holds the quadrotor in a delicate dance of stability. Even small changes to the system, such as propeller damage, can induce loss of control. Not only does this damage limit the capability of the affected propeller, it also generates additional (structural) dynamics and asymmetrically intensifies the already problematic vibrations and sensor noise (47–49). These effects need to be compensated for by the remaining (healthy) rotors. In tandem, the targeted filters designed to limit these issues become less effective, allowing (some of) the problematic dynamics to contaminate the feedback signal. While further clever engineering may in principle accommodate such effects (18, 50), this requires model knowledge that is lacking in the face of unknown system changes.

### Quadrotor Flight Experiments.

**Fig. 2.**
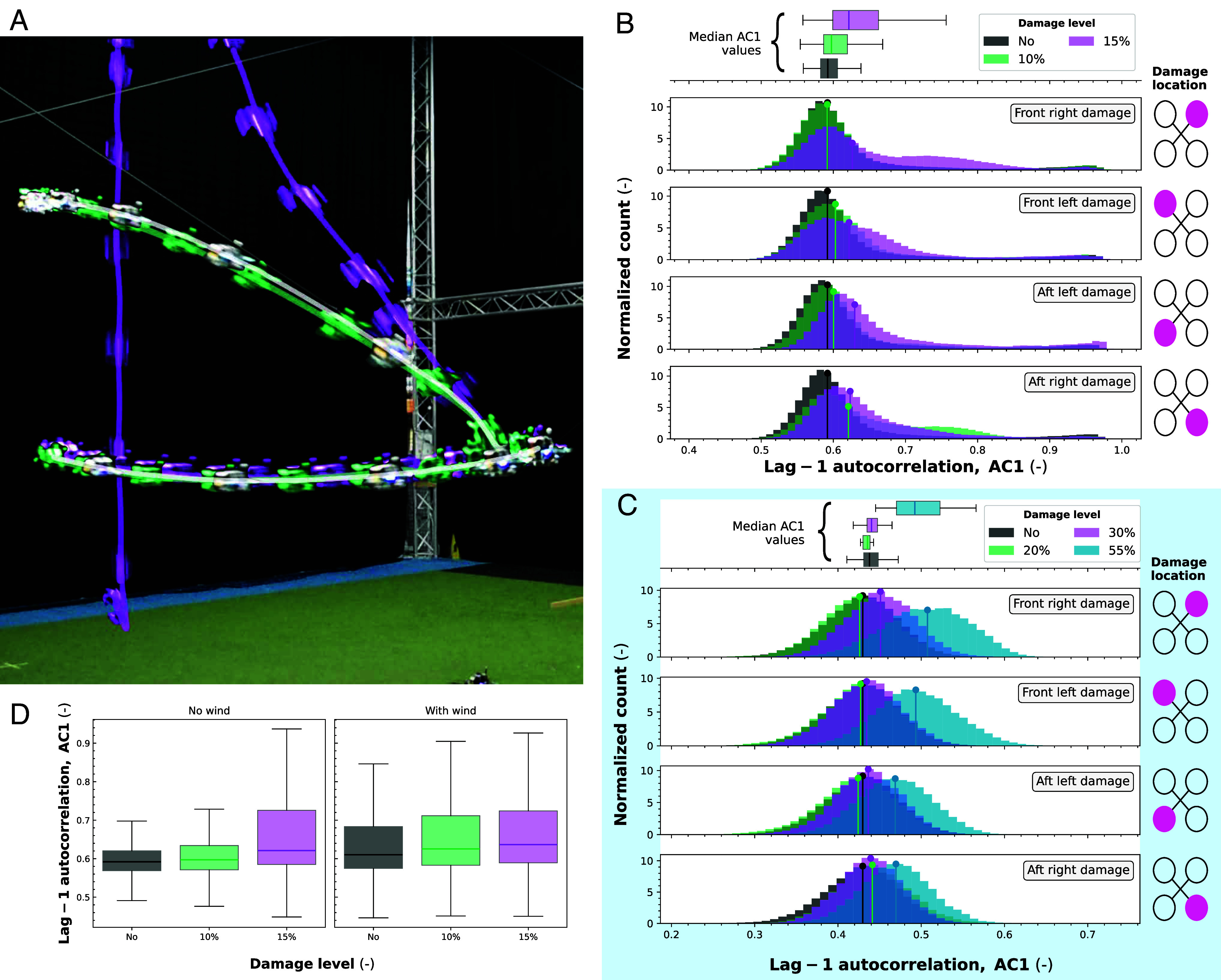
Quadrotor instability due to incremental blade damage. The quadrotor system shifts toward instability (loss of control) with increasing propeller damage. The composite image (*A*) depicts the occurrence of such a loss of control event at the 15% damage level (purple silhouette) during flights of the INDIFlight controlled “DragonFly” quadrotor. The presence of this vulnerability remains hidden in the flight performance at the 10% damage level (green silhouette), which closely follows the nominal 0% damage (white silhouette) flight. Nonetheless, this approach to instability is reflected by generic indicators of CSD, as shown by corresponding increases in lag-1 autocorrelation (AC1) metric alongside the damage severity for the INDIFlight controlled DragonFly (*B*). The vertical axis (“Normalized count”) denotes the number of AC1 values in each histogram bin, normalized such that the total area under the histogram equals one. For each damage location, the depicted AC1 distributions are composed of the AC1 values combined across all four rotor measurements (i.e., including the undamaged rotors) and flight conditions. Moreover, the characteristic increase in AC1 alongside damage severity is also observed for a similar quadrotor that runs a more (blade) damage resilient controller (Betaflight 4.3.2), albeit tolerant of a higher damage degree (*C*). Though the extent of the AC1 increase depends on the location of the damage and controller type, both *B* and *C* show a consistent increase in AC1 alongside the damage level across all damage locations. This is reflected in the corresponding boxplots that summarize the median AC1 values aggregated by only damage level. Furthermore, the increase in AC1 due to damage persists despite the presence of wind disturbances, as indicated by medians and interquartile ranges of the box plots in *D*.

The adverse effects of propeller damage can result in “flyaway” loss of control events (i.e., uncontrolled ascending flight provoked by rapid and unstable oscillations in rotor speeds, see Fig. 2*A* and Movie S1). These flyaways occur consistently near a critical damage level of 30% (Movie S2) for our autonomous INDIFlight^*^ controlled quadrotor, named DragonFly (Fig. 1*A*). Though the INDIFlight controller can maintain stability below the critical 30% damage threshold, loss of control occasionally occurs already at 15% damage in demanding operating conditions (such as Fig. 2*A*).

**Fig. 3.**
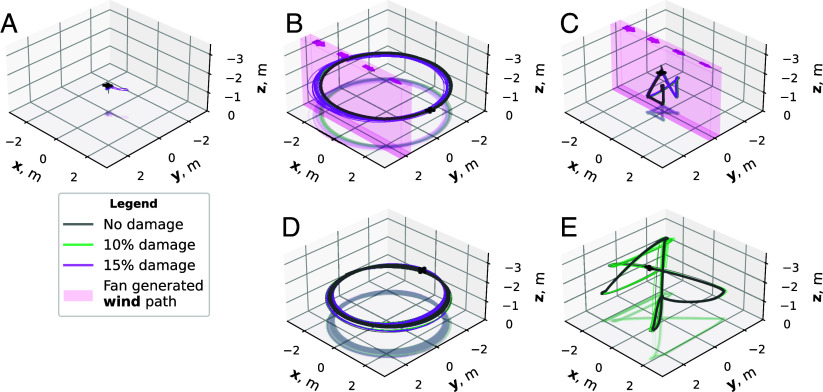
Autonomous flight trajectories. Illustration of the various flight trajectories flown during the experimental campaign of the INDIFlight controlled DragonFly. (*A*) depicts a set of trajectories of the hovering flight task at each damage level. Examples of low intensity flight tasks are shown in (*B*) and (*C*) whereas flight trajectories with more demanding maneuvers are depicted in (*D*) and (*E*). These include circular tracking flights at 1.5 m/s (*B*) and 3.0 m/s (*D*) and “small” (*C*) and “large” (*E*) rectangle vertex set point tracking tasks. Moreover, (*B*) and (*C*) involve both windless and windy flights, wherein the region of influence and direction of the wind (when active) is highlighted by the shaded region. In all plots, the colors denote the damage level, and the opaque lines depict the projection of these trajectories on the x–y plane for spatial context.

Therefore, to monitor this approach toward instability, we conduct flight experiments with the DragonFly subject to 0, 10, and 15% blade damage levels (Fig. 1*C*). These flight experiments involve both hovering and trajectory tracking tasks (Fig. 3) in either windless or windy conditions to reflect real world operations (more details on data collection can be found in *Materials and Methods*). Furthermore, to limit coincidence, we conducted multiple flights at each damage level and damage location combination. All these flights (n = 247) are summarized in *SI Appendix*, Table S1.

**Fig. 4.**
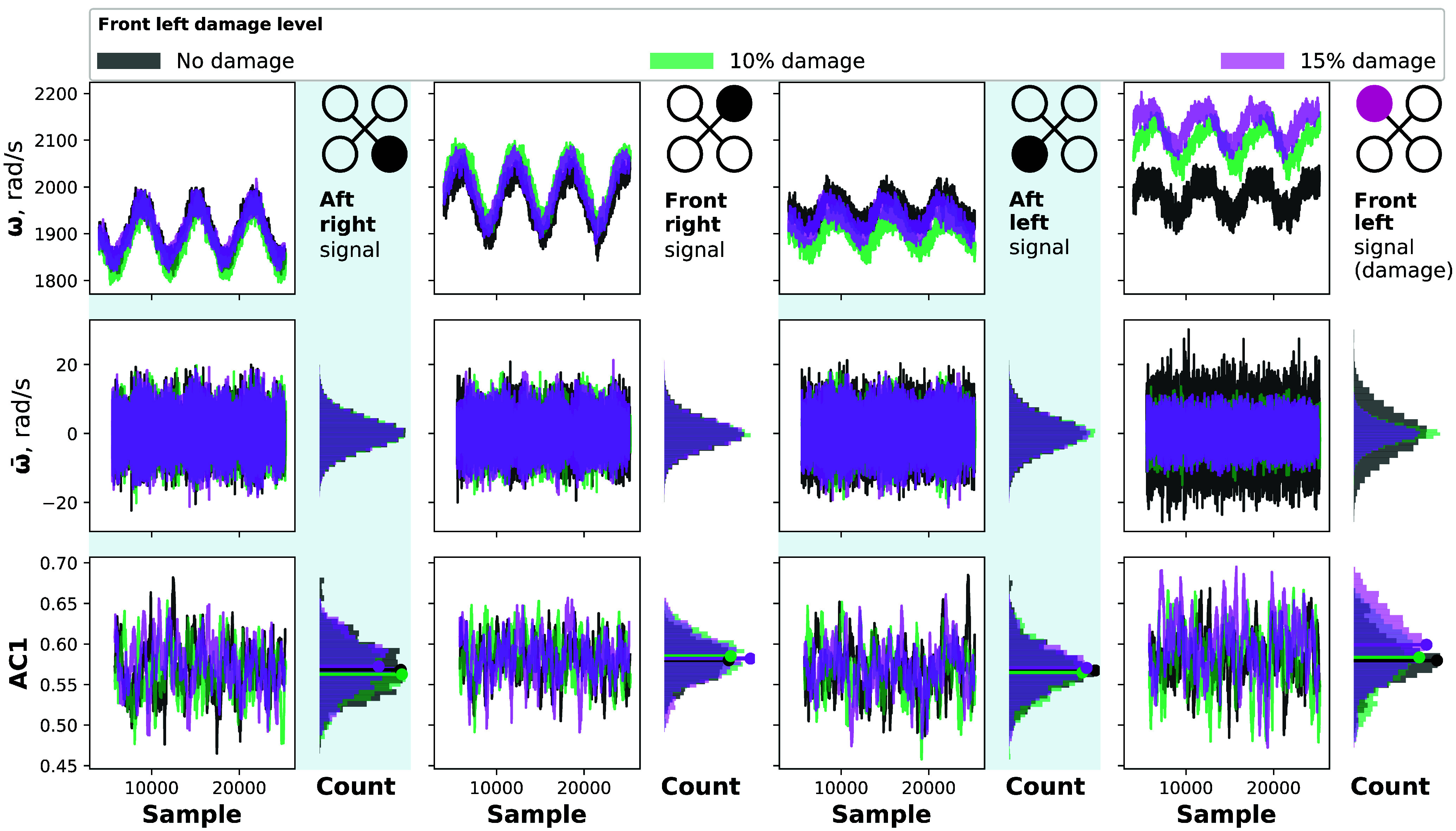
Construction of the generic indicators of quadrotor instability. The generic indicators of quadrotor instability are derived from rotor speed measurements, *ω*, (top row of plots) obtained from the DragonFly quadrotor’s onboard electronic speed controller. Slow trends in these measurements are removed using a moving average detrender (window size of 3 samples, or 0.006 seconds), shown in the middle row of plots. This transforms the detrended rotor speeds, $\overset{¯}{\omega }$, into an autoregressive process upon which the lag-1 autocorrelation metric (AC1) of CSD may be applied. The AC1 is calculated over a moving window of 300 samples (equal to 0.6 s) sliding over each of the detrended rotor speed signals individually. The resultant AC1 are depicted in the plots along the bottom row. The median AC1 values are highlighted in the accompanying count histograms. Data for these plots come from three individual windless circular tracking flights of the DragonFly at a speed of 1.5 m/s: i) no damage to the front left rotor, ii) 10% damage to the front left rotor, and iii) 15% damage to the front left rotor. Measurements from all rotor locations (including undamaged rotors) are used to inform the generic indicators. The columns correspond to the different rotor locations where the solid circle indicates the propeller location from which the measurement is obtained. The damaged rotor (front left) is denoted by the pink circle in the rightmost column.

For each flight, we quantify CSD through the lag-1 autocorrelation metric (AC1) (28, 51) along a sliding window applied to the rotor speed measurement timeseries. The AC1 are calculated over a sliding window to form a persistent monitor of instability suitable for real-world operations. Though we explicitly damage the quadrotor here, in reality, damage may accumulate during operation or between missions (e.g., during transportation). Here, the AC1 timeseries are calculated for each of the (detrended) rotor speed measurements individually in order to observe the holistic (i.e., net) effect of the damage. A detailed treatment of the generic indicator construction process is provided under *Materials and Methods* alongside an illustration of this procedure in Fig. 4.

These early warning indicators reveal how the quadrotor’s loss of stability as the blade damage grows is reflected by subtle changes in dynamics consistent with CSD. Aggregating the early warning indicators (i.e., the AC1 values) by damage level across flight tasks shows a clear increase in AC1 alongside the degree of blade damage from (nominal) 0% damage to 15% damage (Fig. 2*B*). This general trend is consistent across all damage locations. Likewise, for all damage locations, the AC1 distributions display long tails of high AC1 values that suggest short periods of near instability. These coincide precisely with brief moments of aggressive maneuvering for which the proximity to instability only becomes apparent under severe enough blade damage that “pushes” the system beyond the stable regime (as occurs in Movies S1 and S4).

**Fig. 5.**
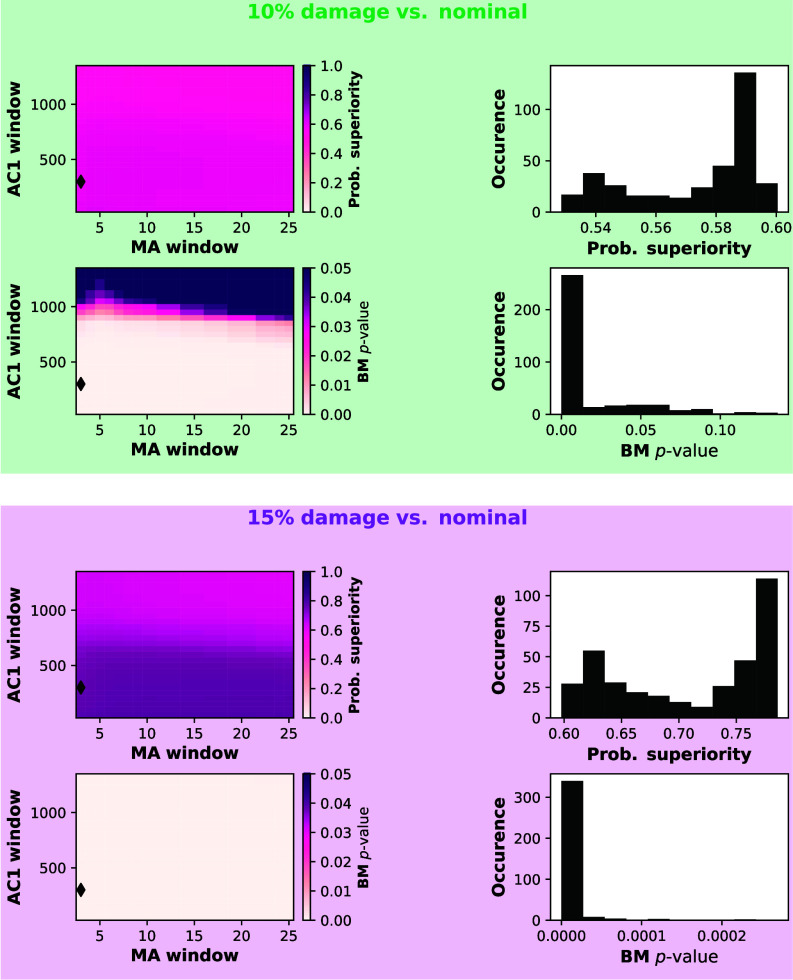
Sensitivity of statistical results (median). The generic indicators of instability are calculated over a moving average (MA) detrender window and lag-1 autocorrelation (AC1) window. The sensitivity of the significance—as assessed by a one-sided Brunner Munzel (BM) test—of increases in AC1 (due to 10 and 15% propeller damage) with respect to variations in these parameters are depicted in the heatmaps along the left column plots, organized by damage degree. The right column plots provide histograms of the associated BM statistical test p-values and probability of superiority, *PS*, effect sizes.

The general increase in AC1 as a function of the damage level observed in Fig. 2*B* is supported through statistical analyses (see *Materials and Methods* for details). Moreover, the significance of these trends persists despite variations in the generic indicator construction parameters (Fig. 5). Here, we report a few notable results.

A one-sided Brunner Munzel test (52) shows that the increase in median AC1 due to damage, no matter the damage location, is significant at both the 10% (t = 3.70, P<0.001) and 15% (t = 12.45, P<0.001) damage levels (Table 1). The estimated probability of superiority (i.e., Vargha Delaney A) is PS = 0.59 (95% CI: [0.54, 0.63]) at the 10% damage level and PS = 0.76 (95% CI: [0.72, 0.80]) with 15% damage. This indicates that blade damage consistently and strongly increases the underlying AC1 distribution, which is consistent with the qualitative increase in loss of control events observed at the 15% damage level (e.g., Fig. 2*A* and Movies S3 and S4).

**Table 1.** Statistical analyses (median)

Damage comparison	Num. *A*, Num. *B*	PS, P(*A*>*B*) [95% CI]^†^	HL shift [95% CI]^‡^	HL / IQR(*B*)	BM *P*-value^§^
10% dmg (*A*) > No dmg (*B*)					
All rotors	420, 200	0.59 [0.54, 0.63]	6.1e-3 [2.8e-3, 9.6e-3]	0.26	<0.001
Front rotors only	220, 200	0.54 [0.49, 0.60]	2.9e-3 [−8.0e-4, 6.6e-3]	0.12	0.064
Aft rotors only	200, 200	0.64 [0.58, 0.69]	1.0e-2 [5.9e-3, 1.5e-2]	0.43	<0.001
Right side rotors only	260, 200	0.57 [0.52, 0.62]	4.8e-3 [1.2e-3, 8.7e-3]	0.21	0.005
Left side rotors only	160, 200	0.62 [0.56, 0.68]	8.2e-3 [4.0e-3, 1.2e-2]	0.35	<0.001
15% dmg (*A*) > No dmg (*B*)					
All rotors	368, 200	0.76 [0.72, 0.80]	2.7e-2 [2.1e-2, 3.3e-2]	1.14	<0.001
Front rotors only	196, 200	0.70 [0.65, 0.75]	2.1e-2 [1.5e-2, 3.0e-2]	0.91	<0.001
Aft rotors only	172, 200	0.83 [0.79, 0.87]	3.1e-2 [2.5e-2, 3.6e-2]	1.31	<0.001
Right side rotors only	228, 200	0.76 [0.71, 0.81]	2.3e-2 [1.8e-2, 2.8e-2]	0.98	<0.001
Left side motors only	140, 200	0.76 [0.71, 0.82]	3.5e-2 [2.7e-2, 4.4e-2]	1.51	<0.001
Aft (*A*) > Front (*B*)					
10% damage	200, 220	0.60 [0.54, 0.65]	8.5e-3 [3.7e-3, 1.4e-2]	0.33	<0.001
15% damage	172, 196	0.57 [0.51, 0.63]	9.5e-3 [1.8e-3, 1.7e-2]	0.12	0.010
Left (*A*) > Right (*B*)					
10% damage	160, 260	0.54 [0.49, 0.60]	3.6e-3 [−1.1e-3, 8.4e-3]	0.12	0.066
15% damage	140, 228	0.55 [0.49, 0.62]	8.6e-3 [−1.0e-3, 1.9e-2]	0.17	0.044

Statistical analyses for increases in lag-1 autocorrelation medians due to the presence of rotor damage.

^*^PS = Probability of superiority, CI = Confidence interval, HL = Hodge’s Lehman shift, IQR(B) = Interquartile range of B, and BM = Brunner-Munzel. The median AC1 distributions being compared are denoted by A and B, respectively, where B acts as the “control” for the BM statistical test

^†^PS computed using half credit for ties (i.e., where rank A = rank B). The associated CI are obtained via Wald’s CI.

^‡^The HL shift CI obtained through an asymptotic approximation to the U-statistic.

^§^The BM test is conducted with the one-sided alternative that A > B.

Furthermore, though the quadrotor is often considered symmetric, our AC1 statistical analyses reveal hidden asymmetries in its stability. For example, the increase in AC1 due to aft rotor damage is significantly higher than front rotor damage at both the 10% (t = 3.56, P<0.001) and 15% (t = 2.35, P=0.001) damage levels (Table 1). Similarly, 15% damage to the left rotors tends to increase the AC1 significantly more than damage to the right rotors: t = 1.71, P=0.044 (Table 1).

These curious asymmetries emerge due to system specific nuances, despite a seemingly consistent design. For instance, the front-aft asymmetry is most likely due to the rear placement of the battery on the quadrotor (Fig. 1*A*), which pulls the center of gravity backward. This demands more effort from the two aft rotors. Likewise, the left–right asymmetry is a function of the varying construction quality of the motors and propellers (48, 49). On the DragonFly, the aft left motor runs 5 ^°^C warmer than the other motors, indicating a fault or damage. Such characteristic system traits have a clear effect on system stability (Movie S3) but are challenging, perhaps even impossible, to model effectively. Nonetheless, their net effect on stability can effectively be monitored through our dynamical indicators of resilience.

To further demonstrate this holistic monitoring ability, we conduct additional incremental damage flight experiments with a similar quadrotor (Fig. 1*B*) that runs a more damage tolerant flight control software: Betaflight. By forfeiting autonomous capabilities, this controller relies less on the sensors (specifically, the accelerometers) which are most affected by the propeller damage induced oscillations. Hence, the Betaflight controlled quadrotor can hover at damage levels of 55%, unlike the 30% limit of its INDIFlight counterpart. This improved robustness is reflected in the associated AC1 distributions (Fig. 2*C*). Although the INDIFlight controller may be modified to also improve its resilience to blade damage, doing so simply shifts the problem of instability to a higher damage level. With or without these improvements, the generic indicators discussed here remain sensitive to shifts in stability.

This ability to monitor for loss of stability extends to more realistic stochastic operating scenarios that quadrotors face in-the-field. Fig. 2*D* reveals that, for the INDIFlight controlled DragonFly, the growth in AC1 due to blade damage occurs even under the presence of both gust-like and turbulent wind (relevant trajectories in Fig. 3 *D* and *E*, respectively). However, it becomes challenging to distinguish between increases in AC1 arising from loss of stability due to moderate damage (e.g., 10%) and increases resulting from a changed stochastic regime (51) of the wind. Indeed, CSD often struggles to detect approaching transitions in highly stochastic environments (53, 54). Nonetheless, the near identical characteristics of the 15% damage level AC1 values in Fig. 2*D* show that, once the damage becomes severe enough to dominate controller behavior, loss of stability is observable through our generic indicators of resilience despite the stochastic wind effects. This is due to the choice of the rotor speeds (i.e., controller action) as the basis for the indicators, which directly captures the controller behavior. Therefore, while these windy experiments are a simplification of reality, these results suggest that our generic indicators can sense significant loss of stability despite the presence of (moderate) stochasticity.

## Outlook

Our quadrotor flight experiments demonstrate that CSD can be used to detect loss of stability in controlled systems. This is achieved solely through measurements obtained from the low-cost onboard components, making the approach suitable for direct use both in-the-field and across systems equipped with similar sensors. This opens up the possibility to monitor resilience of dynamic systems on the fly, rather than compute it upfront from models that can become inaccurate in the face of incremental damage or other problems. For example, many engineered systems suffer component degradation over their life cycle, may harbor hidden inconsistencies in manufacturing quality, and can behave differently depending on environmental conditions (e.g., dry vs. wet road surfaces). A new generation of controllers based on our findings may be designed to monitor for hidden changes in stability through CSD and leverage this information to warn in much the same way that we perceive pain: if our ankle hurts, we may not know why it hurts, but we are aware that we should be careful as something is different from normal. Depending on the severity of the shift in stability, the new class of controllers may then advise an intervention to maintain safety. Thus, CSD equips the controller of a feedback system with awareness of changes to its safe operating space, bridging the gap between internal models and reality.

While our approach offers (early) warning of a nearing instability, it does not directly give an indication of how “far”; away the critical threshold is. Likewise, the source(s) of the instability remains unknown. Therefore, it is challenging to determine from the indicator alone how to prevent the loss of stability. Moreover, our dynamic indicator is a relative metric in the sense that we require a reference for nominal system behavior. Therefore, if the nominal system itself is already dangerously close to instability, then the system may reach its critical threshold before any meaningful shifts in stability can be observed.

Furthermore, while our approach is generic, it relies on measuring informative variables to observe meaningful changes in stability. Identifying such variables remains an open challenge for practical use in many real systems. In general, we view variables which relate to the output of the controller and input to the “uncontrolled” system as prime candidates (take, for example, the rotor speed measurements for the quadrotor). Even so, some systems may be limited in their observability of these variables, especially at the necessary fidelity (i.e., accuracy and sampling rate).

Despite such limitations, the feasibility of real-time monitoring of the inherent stability of engineered systems demonstrated here has profound practical implications. Those include the possibility to warn for dangerous instability, prompting operators to halt use of the system for inspection and maintenance. Likewise, these indicators may be used in a quality control setting during production and manufacturing to identify anomalies. An entirely different application of our approach is to use it as a design tool allowing one to “tinker” with engineered systems and measure immediately whether small adjustments are an improvement when it comes to stability. More speculative, automated resilience monitoring may allow systems to diagnose issues during operation and guide real-time reconfiguration in a way that improves resilience thus adapting to changing environmental conditions or system damage. To this end, we may draw inspiration from the natural world to explore and guide such real-time adaptive behavior in engineered systems.

## Materials and Methods

### Quadrotor Flight Data Collection.

Flight experiments are conducted with two quadrotors built using similar off-the-shelf commercial components (*SI Appendix*, Table S2), but operated using two different flight controllers (i.e., software). The main results of incremental blade damage are obtained through the autonomous drone named DragonFly (Fig. 1*A*), which runs the INDIFlight^†^ controller. Conversely, the “HoverFly” quadrotor (Fig. 1*B*) operates the nonautonomous Betaflight 4.3.2^‡^ controller and is used to demonstrate how closed-loop instability is a function of the controller, a trait that is reflected through the generic indicators of instability.

#### Flight control software.

A near “open-loop” control policy has been adopted by Betaflight, affording flexible flight to hobbyist human pilots. While desirable for drone racing and freestyle flight, the low-level control managed by Betaflight leaves the human pilot largely responsible for maintaining stability. Consequently, Betaflight does not natively support autonomous flight. In contrast, INDIFlight extends the base Betaflight infrastructure to enable autonomy by relying on additional sensors. Namely, the onboard accelerometer measurements facilitate thrust control while an external motion capture system (OptiTrack) is used to measure the quadrotor’s position, velocity, and orientation (Fig. 1*F*).

However, it is these additional feedback measurement dependencies that provoke instability within INDIFlight when operating with propeller damage. The accelerometer is sensitive to excessive vibrations—which intensify with growing damage—that cause aggressive overcorrections from the controller, leading to instabilities (following the mechanisms described in *SI Appendix*, Example 2). Note that although one may design targeted filters to mitigate this sensitivity, doing so only postpones the issue of instability; at a certain damage level, the quadrotor is unable to fly irrespective of the filtering applied.

Moreover, the transmission of the external motion capture data also affects closed-loop stability due to interference and congestion in the 2.4 GHz WiFi communication link between the ground station and quadrotor (Fig. 1*F*). The result is an inconsistent transmission rate of 100 to 200 Hz with occasional dropouts. In contrast, the control loop of INDIFlight runs onboard at 1,000 Hz and therefore holds the position and attitude measurements until a new measurement update is available. This can result in mismatches between the controller’s belief of the current state and the true state of the quadrotor.

We emphasize that the motion capture facility is only used to facilitate repeatable autonomous flight experiments. Measurements from this system are thus not used when constructing the generic indicators of resilience. That is, extremely accurate or specialized sensor information is not a prerequisite for compatibility with our approach.

#### Autonomous flight experiments.

Autonomous flights of the INDIFlight controlled DragonFly consist of both hovering and trajectory tracking tasks with and without propeller damage (Fig. 3). The trajectory tracking tasks involve following a circle of 3 m radius at airspeeds of 1.5 m/s and 3.0 m/s and maneuvers between the vertices of a three-dimensional rectangle in space, designed to excite each of the four motors individually at least once during the flight. Furthermore, some trajectories are flown in both windless and windy conditions to challenge the controller with further perturbations. Here, wind is generated by a large fan (*SI Appendix*, Fig. S1) which disturbs the quadrotor along its flight path. Adequately rejecting this disturbance can become demanding under propeller damage.

The DragonFly is unable to maintain stable flight at a damage level of 30% (Movie S2). Thus, on approach to this threshold, flight data are collected at damage levels of 0, 10, and 15% at each rotor location separately. We do not collect data on simultaneous rotor damage in order to isolate damage effects to a particular location and investigate how this location may asymmetrically affect the stability of the quadrotor, if at all. Each flight condition — that is, combination of damage level, damage location, and flight tracking task—is summarized in *SI Appendix*, Table S1. Note that some flight conditions could not be completed reliably as they resulted in frequent flyways (such as Movie S3), leading to fewer than five successful flights for the condition. In total, 247 individual flights, lasting around one minute each, are completed without a crash occurring.

We rely on the rotor speed RPM measurements, *ω*, as the basis for our dynamical indicators of quadrotor instability. This is because *ω* serves as both the input to the quadrotor system and the (motor dynamics filtered) output of the controller (*SI Appendix*, section S2). As such, *ω* captures the potentially unstable interaction between the controller and the quadrotor. We measure *ω* through the onboard electronic speed controller (ESC) via the Bidirectional DShot protocol. These data are logged at 1,000 Hz. Since INDIFlight applies less filters to the raw sensor data prior to logging than Betaflight, we first apply a 100 Hz low-pass fourth-order Butterworth filter to limit the power of high-frequency noise in the measurements to approximate the low-pass filtering conducted by Betaflight prior to data logging. Subsequently, we downsample the INDIFlight measurements to match the 500 Hz logging rate of Betaflight. These filtering steps (i.e., 100 Hz low-pass filter and downsampling) result in an INDIFlight rotor speed frequency spectra that is similar to Betaflight, affording the use of consistent CSD parameters across the two systems.

#### Human-piloted flight experiments.

The piloted flight tests of the Betaflight controlled HoverFly serve as means to compare propeller damage tolerance under different flight control software. Due to a lack of autonomous capabilities, only hovering flights are conducted to promote consistency in the pilot’s behavior. Five hovering flights, lasting around one minute each, are flown at each damage level (from 0 to 55% damage as shown in Fig. 1*C*) and each rotor location. As with the INDIFlight controlled DragonFly, only one propeller is damaged at a time. Although the hovering flight task is less demanding than the autonomous maneuvers conducted with the DragonFly, manual hovering flight with the DragonFly is already impossible at the 30% damage level due to persistent flyaways (such manual flight tests with INDIFlight are shown in Movie S2). Thus, the improved resilience of the Betaflight controlled HoverFly is not due to the less demanding maneuvers alone but is primarily a function of the controller itself. In total, 120 flights of the HoverFly are flown for which the rotor speed RPM measurements are provided by the onboard ESC via Bidirectional DShot, logged at 500 Hz.

### Generic Indicators of Instability.

CSD is used as an early warning of critical transitions across a family of bifurcations (28, 40). At these bifurcation points, the dominant eigenvalue, $\lambda_{\max}$, of the system’s Jacobian matrix exchanges stability. Mathematically, the real part of this eigenvalue approaches zero (i.e., $\left|Re\left(\lambda_{\max}\right)\right|\to 0)$ near the bifurcation point and, as a consequence, the rate of recovery of the system to equilibrium slows down. It is this change in rate of recovery that CSD is sensitive to. Analogously to bifurcations, as a controlled system (locally) approaches instability, the dominant eigenvalue of the associated closed-loop (i.e., combination of system and feedback controller) Jacobian also approaches zero: Re($\lambda_{\max}$)→0. Therefore, CSD is well equipped to monitor shifts toward instability of nominally stable (controlled) systems.

To this end, we employ the lag-1 autocorrelation (AC1) metric of CSD as our generic indicator of controlled system instability. The motivation for this is twofold. First, as a correlation metric, the AC1 enjoys a well-defined domain: $AC1\in \left[-1,1\right]$. Second, the increase in AC1 as a critical transition is approached (i.e., AC1 →1) is often consistent (51). These characteristics facilitate a generic comparison between variations of the same controlled system nearing a critical transition.

We calculate the AC1 for the quadrotor using each of the rotor speed measurements, *ω*, since instabilities driven, in part, by the controller are observable through *ω* (*SI Appendix*, section S2). This choice of early warning variables relies only on onboard measurements, making our approach deployable for quadrotors “in-the-field” and compatible with other dynamical systems reliant on similar low-cost sensors and electronics as an “add-on” safety filter. As such, the fundamental approach toward constructing these generic early warning signals based on controller outputs is suitable for a vast array of complex controlled systems—ranging from self-driving cars and aircraft to industrial robots—that can each approach instability.

#### Continuous instability monitor.

The generic indicators are calculated along a moving window sliding over the flight data timeseries—rather than over nonoverlapping segments of flight—to reflect the intended application of our indicators as a continuous monitor of instability. While we do not compute the indicators in real-time in our experiments, we nonetheless demonstrate its sensitivity for such a task via this rolling window approach. Furthermore, we note that incremental damage may be incurred during operation or between missions (e.g., damage during transportation) for which the rolling window approach is well suited to promptly detect a corresponding shift in stability.

#### Lag-1 autocorrelation per rotor.

For each of the four rotor speed measurements, we first estimate slow trends in the timeseries through a moving average taken within a sliding window along the timeseries. These slow trends are then subtracted from the original signal to detrend it. We chose a moving average filter for its simplicity and suitability for real-time use to detrend new measurement data as it becomes available. Here, we elect a window size of three samples (equivalent to 0.006 s for our data) to arrive at a detrended (i.e., residual) signal which resembles a first-order autoregressive process.

Subsequently, we estimate the AC1 via a sliding window running along each of the detrended rotor speed measurements individually. Within this window, the AC1 coefficient is estimated via a Pearson correlation of the detrended signal with itself lagged by one sample. We heuristically select a window size of 300 samples (equal to 0.6 s) such that the nominal (i.e., no damage) AC1 values are centered near AC1≈0.5. Doing so affords space for shifts in AC1 to be observed.

This objective of detecting shifts in stability due to system variations, rather than detecting the exact moment of tipping itself, motivates our choice to estimate the AC1 via a Pearson correlation instead of the more conventional ordinary least squares estimator. The Pearson correlation ensures that $AC1\in \left[-1,1\right]$, providing a consistent domain mapping datum upon which incremental variations of a (controlled) system can be compared whereas OLS does not provide such guarantees on the bounds of the estimated AC1 coefficient.

An example of how the AC1 timeseries are constructed is illustrated in Fig. 4, wherein the columns delineate the process per rotor. The result is a set of four histograms of the AC1 values observed for each rotor during the flight. This figure shows that most of the increase in AC1 is due to the damaged rotor itself, whereas the undamaged rotors experience limited shifts in AC1. Despite this, to reflect real world operations, we observe the shifts among all rotors simultaneously as the location of the damage is often unknown a-priori (though its location may be diagnosed through our indicator). Therefore, to instead visualize the holistic shift in stability of the quadrotor due to damage (as in Fig. 2), we concatenate the AC1 values derived from each of the four rotors into a single representative AC1 frequency histogram per damage level (e.g., 10%) and damage location (e.g., front right). That is, each histogram in Fig. 2 includes data of all rotor measurements across all the relevant flight tasks (summarized in *SI Appendix*, Table S1). Note that this in fact disadvantages the sensitivity of our indicator for detecting loss of stability.

### Statistical Analysis.

Since the lag-1 autocorrelation (AC1) values of a given flight task are computed over a rolling window, they are (time) dependent. Therefore, for statistical analyses, we collect the median AC1 value of each rotor per flight of the DragonFly (i.e., each flight yields four independent median AC1 values, one from each rotor location). We choose the median as it reflects the center point of the underlying AC1 histogram and is robust against outliers that can occur during flight due to abnormal conditions (e.g., brief WiFi dropouts).

We then aggregate these median AC1 values across damage conditions of interest to form a representative AC1 distribution (see “Damage comparison” in Table 1 for the damage conditions considered in our analyses). As an example, the median AC1 distribution that represents the nominal condition is composed of the median AC1 values of each flight of the DragonFly with 0% damage (first row in *SI Appendix*, Table S1). This yields an AC1 distribution with 200 data points (50 flights with 4 median values each, representing each rotor measurement).

Subsequently, the significance of increases in these median AC1 distributions due to propeller damage is assessed through a (one-sided) Brunner Munzel nonparametric test. This test is used to evaluate the probability, P, that a random sample from a damaged AC1 distribution of interest is larger than a random sample from the reference nominal AC1 distribution, under the null hypothesis that this probability is equal (i.e., P=0.5). The null hypothesis implies that the distributions are stochastically the same. Note that we favor the Brunner Munzel test over the Mann–Whitney U test since the former allows for unequal variances between the two distributions (52), which is the case for our data: the damaged AC1 distributions tend to harbor larger variances than the nominal distribution. These differences arise from nonlinear propeller damage effects between the various maneuvers conducted across the flight experiments. We consider a result significant if the Brunner Munzel *P*-value is less than 5% (i.e., P<0.05). A natural effect size measure for the Brunner Munzel test is to evaluate the Probability of Superiority (Vargha-Delaney A), PS, which quantifies how likely it is that a random AC1 sample from distribution A (e.g., damaged) is greater than a random AC1 sample from distribution B (e.g., nominal):

$$PS=P\left(A>B\right)+\frac{1}{2}P\left(A=B\right)$$

In other words, the PS represents the consistency of increases in AC1 due to propeller damage, relative to the nominal case. Here, we assign half credit in the event of ties between the AC1 values of the damaged and undamaged distributions. Thus, if these distributions are indeed the same (i.e., A = B for all values) then PS = 0.5. We likewise measure the corresponding magnitude of increase in AC1 through the Hodges’ Lehman shift, scaled by the interquartile range of the nominal AC1 distribution to contextualize this effect size. That is, how much has the AC1 increased in comparison to the interquartile range of the nominal AC1 baseline.

The statistical analysis results are summarized in Table 1 and show that damage incurred by the propellers significantly increases the AC1 values. The magnitude of increase grows alongside the damage level. Also shown in Table 1 are the statistical tests between damage locations (i.e., front vs. aft and left vs. right) and the corresponding increase in AC1, revealing significant asymmetries in stability for the DragonFly quadrotor.

#### Indicator parameter sensitivity analysis.

We assess the sensitivity of the statistical results as a function of the generic indicator parameters (i.e. moving average detrender window size and AC1 window size). The moving average window is varied from 3 to 25 samples (0.006 to 0.05 s) and AC1 window from 50 to 1,500 samples (0.1 to 3.0 s). Fig. 5 illustrates the change in Brunner Munzel *P*-value and Probability of Superiority, PS, effect size for different combinations of these generic indicator parameters.

The trends in Fig. 5 indicate that the increases in AC1 due to damage remain largely significant despite variations in the generic indicator parameters, especially at the 15% damage level. Nevertheless, the Brunner Munzel *P*-value and PS deteriorate with increasing AC1 window size. In contrast, variations in the moving average window have little effect.

These trends reflect the fact that the quadrotor is an incredibly fast system. Thus, the dynamics of interest often reside at high frequencies (i.e., short time scales), especially in the face of propeller damage. Nonetheless, these high frequency components can bleed into lower frequencies: instabilities become apparent over numerous cycles of the high-frequency effects. Moreover, these statistical results agree with the qualitative observations of instability for the DragonFly quadrotor already at the 15% damage level (Movie S3).

## Supplementary Material

Appendix 01 (PDF)

Movie S1. Comparison of flight trajectories of the INDIFlight controlled DragonFly quadrotor at the 0% (white), 10% (green), and 15% (purple) damage levels where loss of control occurs at the 15% damage level (front right rotor).

Movie S2. Shows two perspectives of the inability of the INDIFlight controlled DragonFly quadrotor to maintain stable flight at the 30% damage level. Here we attempt to pilot the drone in manual mode (i.e. piloted flight; removing the dependency on the motion capture system). Nonetheless, the rotor speeds are unstable due to excessive vibrations. This can be heard in the video.

Movie S3. Persistent loss of control of the INDIFlight controlled DragonFly quadrotor with the 15% damage to the aft left rotor. The quadrotor is tasked with following vertices of a rectangle.

Movie S4. Aggressive maneuver during trajectory initialization sequence for circular flights. These cause brief periods of ‘near instability’ which remain unproblematic for the No and 10% damaged scenarios but lead to loss of stability during the maneuver for the 15% damage scenario.

## Data Availability

The raw flight data of the propeller damage tests conducted with the DragonFly and HoverFly quadrotors is freely available (55). The raw simulation data of ecological and control systems en-route to critical transitions underlying the results of the SI Appendix is also freely available (56). The code used to process these data sets, generate the main findings of this paper, and reproduce the figures is made open access (57). All remaining study data is included in this article and/or the supporting information.

## Footnotes

^*^INDIFlight is an incremental nonlinear dynamic inversion (INDI) based controller for quadrotors, available at: https://github.com/tudelft/INDIFlight.git.

^†^Freely available here: https://github.com/tudelft/INDIFlight.git.

^‡^Freely available here: https://www.betaflight.com/download.
