# Supplementary material for: Early warning signals for loss of control in complex systems
Source: Proc Natl Acad Sci U S A. 2026 Jul 1;123(27):e2608847123. doi: 10.1073/pnas.2608847123 (PMC13342939; doi:10.1073/pnas.2608847123)
Supplement: Supplementary file 1 — Appendix 01 (PDF) [file pnas.2608847123.sapp.pdf]

Supporting Information for

Early warning signals for loss of control in complex systems

Jasper J. van Beers, Marten Scheffer, Prashant Solanki, Ingrid A. van de Leemput, Egbert H. van Nes, and Coen C. de Visser

Marten Scheffer.
E-mail: marten.scheffer@wur.nl

This PDF file includes:

Supporting text
Figs. S1 to S7
Tables S1 to S2
Legends for Movies S1 to S4
SI References

Other supporting materials for this manuscript include the following:

Movies S1 to S4

Supporting Information Text

S1. Feedback instability examples

Example 1.

Effect of delay in feedback systems. Real systems harbor inherent delays. Consider, for example, the delays in blood pressure regulation after one stands up rapidly or the lag in temperature change that follows the adjustment of a thermostat. These delays can induce instability in 'closed-loop' systems (i.e. controlled systems for which the controller's actions depend on the system outputs). We illustrate this using a simple second order (linear) system (eq. 1), though the destabilizing effect of input-output delay extends to real-world nonlinear systems. Equation **??** is expressed in the Laplace domain with s the complex Laplace variable, ω_n denotes the natural frequency of the system and ζ is its damping ratio. Let $\omega_n = 1$ and $\zeta = 0.5$.

$$H(s) = \frac{\omega_n^2}{s^2+2\zeta\omega_n s+\omega_n^2} \quad [1]$$

Figure S2 **a** illustrates the inherent delay of $P = H(s)$ in 'open-loop' (i.e. inputs, denoted by the dashed line, made by the controller are invariant of the system output, given by the solid line). If this delay is known, it can be compensated for in the control design to achieve the desired tracking behavior. For example, we design eq. 2 for near perfect tracking of the input signal, seen in fig. S2 **b** with $P = H(s)$.

$$C(s) = 4.5 + \frac{0.5}{s} \quad [2]$$

However, additional delays can arise in real-world systems, for instance, due to actuator dynamics and faults, time-varying dynamics, natural component degradation or even environmental conditions that may limit the effectiveness of the controller.

In this example, we consider the presence of first order actuator dynamics (eq. 3) which increase the delay of the open-loop system as shown in fig. S2 **c** with $P = H_{act}(s)\, H(s)$.

$$H_{act}(s) = \frac{1}{2s+1} \quad [3]$$

Then, under the same control law (eq. 2) the closed-loop system now becomes unstable, depicted in fig. S2 **d** with $P = H_{act}(s)\, H(s)$. This is because a positive (i.e. self-reinforcing) feedback loop in the input-output relation arises; the controller attempts to regulate the system's response but only ends up amplifying it.

Example 2.

State measurement imperfections. Real-world feedback controllers rely on sensor measurements of the system states for control. These sensors are often imperfect and contaminate the measurement signal with artifacts that the controller should ignore, such as biases, noise, additional (sensor) dynamics and sensitivities. Fortunately, these sensor imperfections can be mitigated through clever and targeted filtering. This equips the controller with authority around the frequency ranges of interest and no (or limited) authority around problematic frequency regions (e.g. resonance). While these filters work as desired for the nominal system, issues can re-emerge once the system dynamics change (e.g. due to faults). Similarly, additional delays incurred by the system - which alone are perhaps unproblematic - can destabilize the system when used in tandem with the nominal filters.

We illustrate such a destabilizing effect using the same controlled system as SI Text Example 1 (i.e. eq. 1 and eq. 2) which is now driven by a faster first order actuator (eq. 4) and relies on a sensor (eq. 5) for state feedback.

$$H_{act}(s) = \frac{1}{0.1s+1} \quad [4]$$

$$H_{sens}(s) = \frac{4.5^2}{s^2+1.35s+4.5^2} \quad [5]$$

The individual effects of input-output delay and sensor resonance do not result in instability (fig. S3 **b** and **c** respectively). However, their combined effect does (fig. S3 **d**).

Note that similar resonant sensor dynamics of form eq. 5 are common of many micro-electro-mechanical systems (MEMS), such as those typically found on a quadrotor (1–3). These resonant modes can get excited by vibrations from propeller damage, deteriorating the quality of the feedback signal.

S2. Quadrotor dynamics

While our generic indicators of instability do not depend on a system model, we nonetheless provide the kinematic equations of the quadrotor here to: (i) motivate the choice of the rotor speeds as the early warning variables, and (ii) provide context on the nonlinear dynamics underlying the quadrotor. The quadrotor rigid body dynamics can be written as:

$$\begin{bmatrix} \dot{\mathbf{V}} \\ \dot{\mathbf{\Omega}} \end{bmatrix} = \begin{bmatrix} R\mathbf{g} - \mathbf{\Omega} \times \mathbf{V} + \frac{1}{m}\left(\mathbf{F} - \kappa \sum_{i=1}^{4} \omega_i^2 \mathbf{z_B}\right) \\ I_v^{-1}\left(-\mathbf{\Omega} \times I_v \mathbf{\Omega} + \mathbf{M} + J(\omega \cdot \omega^{\mathbf{T}})\right) \end{bmatrix}$$

where $\mathbf{V}$ denotes the quadrotor's translational velocity and $\mathbf{\Omega}$ describes its rotational velocity, both expressed in the body front-right-down coordinate reference frame (see fig. S4). The transformation between this body frame and the inertial north-east-down (NED) reference frame is captured by rotation matrix R. $\mathbf{g}$ represents the gravity vector in the NED frame. The quadrotor mass and vehicle moment of inertia are denoted by m and I_v respectively. The thrust produced by the rotation of the propellers acts in the negative $\mathbf{z_B}$ direction (see fig. S4) and is described by $\kappa \sum_i^4 \omega_i^2$, where ω_i is the rotational speed of the ith propeller and κ is the thrust coefficient. Likewise, the torques produced by the rotor speeds, $\omega = [\omega_1, \omega_2, \omega_3, \omega_4]^T$, is given by $J\left(\omega \cdot \omega^T\right)$ with J the (torque) control allocation matrix. Finally, $\mathbf{F}$ and $\mathbf{M}$ describe any additional (nonlinear) forces and moments acting on the quadrotor body, for example, due to rotor interaction effects or the presence of attached loads. Indeed, the nonlinear dynamics underlying $\mathbf{F}$ and $\mathbf{M}$ are highly system and application dependent. Nonetheless, our generic indicators of instability may be applied as they are not model dependent.

To this end, we base these indicators on the rotational speed measurements of the propellers (i.e. ω). This is because ω serves as the (general) input to the quadrotor system and describes the (motor dynamics filtered) output of the controller.

S3. Relation to control theory

In this section, we show that critical slowing down (CSD) naturally aligns with control theoretic notions of robustness and reachability. In particular, a decrease in resilience as measured by dynamical indicators of CSD corresponds to a decline in robustness and shrinkage in the backward reachable set. This evidence supports the use of CSD as a generic (i.e. system model-free) indicator to complement traditional means of assessing control system robustness.

To illustrate this link more concretely, consider the following forced mass spring damper system:

$$m\ddot{x} + d\dot{x} + kx = u \qquad [6]$$

where m, d, and k are positive constants (see SI Text: Actuated mass spring damper), x denotes the position and u the forcing input, provided by an actuator. An actuator is used here to introduce realistic delay into the system. Such delay makes this controlled system unstable if the controller becomes too aggressive (through self-reinforcing feedback back loops as described in SI Text: Example 1). We adopt first order actuator dynamics for simplicity:

$$\dot{u} = -\frac{1}{\tau}u + \frac{1}{\tau}\widetilde{u} \qquad [7]$$

In eq. 7, τ denotes the actuator rate constant (i.e. how fast the actuator is) and $\widetilde{u}$ represents the input commanded by the controller:

$$\widetilde{u} = -Kx \qquad [8]$$

with $K > 0$ a constant denoting the controller gain (i.e. how aggressive the controller's response to state changes is). Thus, the objective of this controller is to regulate the position of the system to zero (i.e. the equilibrium of the system).

Generic indicators of instability. The feedback gain, K, commanding the actuated mass spring damper system can be considered a critical parameter since its value governs the stability of the controlled (i.e. closed-loop) system. We note that while linear systems do not experience bifurcations (in the sense that there is no 'branching' of equilibria (4)), they can nonetheless exhibit symptoms of critical slowing down (CSD): as a controlled system approaches instability, the real part of at least one (locally linearized) eigenvalue approaches zero. This is the characteristic phenomenon that results in the slowing recovery rate of a nonlinear system experiencing CSD as a bifurcation is approached.

For the chosen values of m, k, d, and τ given in SI Text: Actuated mass spring damper, the controlled system becomes unstable when $K > 4.59$ as the real part of the system's dominant eigenvalues become positive. We select four discrete gain values on approach to this instability: $K = \{0.5,\ 1.0,\ 1.5,\ 3.0\}$. Then, for each gain value, the behavior of the controlled system is simulated with stochastic perturbations (details in SI Text: Actuated mass spring damper). For each simulation, we calculate the lag-1 autocorrelation (AC1) metric of CSD using a sliding window along each state variable measurement. Further details on how the AC1 values are calculated are outlined in SI Text: Indicators of instability.

The characteristic increase in the AC1 as the controlled system approaches instability due to the incremental amplification of the feedback gain, K, is visible in fig. S5 **a**. This step-wise increase in AC1 mirrors that of a well-studied ecological system with alternative stable states (eq. 14) that also experiences CSD (5) as it approaches a bifurcation through increments of its critical parameter (see fig. S6 **a**). These results, taken alongside those of the quadrotor, show that controlled systems can exhibit behavior consistent with CSD and generic indicators of resilience are capable of detecting approaching instabilities in such systems.

Quantifying resilience to perturbations. Through the lens of control theory, the notion of a system's proximity to instability is often referred to as system 'robustness'. One such tool to quantify robustness in the linear setting is via the so-called 'disk margin' (DM) (6), which assesses how much variation in the closed-loop system's gain and phase (i.e. magnitude and delay, respectively) can be tolerated before it becomes unstable. In other words, the disk margin evaluates how much perturbation is needed to destabilize the system. These perturbations may stem from (uncertainties in) either the controller or the system dynamics itself, such as signal attenuation, amplification, or delays. A larger disk margin indicates that the system can endure greater perturbations and is thereby more robust (i.e. resilient).

We compute the disk margin for the actuated mass spring damper system subject to the incremental amplification of feedback gains ($K \in \{0.5,\ 1.0,\ 1.5,\ 3.0\}$) towards instability. Indeed, the disk margin decreases as the instability is approached (see fig. S5 **b**). Crucially, these results agree with the trends reported by the generic indicators; the AC1 values increase as the gain is amplified. In fact, the disk margin reveals that the closed-loop system can already suffer instabilities at a gain of $K = 3$ (DM $= 0$) if there is any uncertainty. At this gain value, any additional delays in the system (e.g. if the actuator is even marginally slower than expected) will destabilize it. While the disk margin is undoubtedly a powerful analysis tool in this regard, it relies on a linear system model which limits its practicality for real (nonlinear) systems.

To this end, our results suggest that the generic AC1 may be used as a complement to the disk margin in practice. For instance, the AC1 can monitor for shifts in stability on the real-world system in real-time where loss in stability may arise due to, for example, inaccurate or changing system dynamics. A detection of instability, should it occur, would imply a change in (local linear model) disk margin and thus prompts caution and further investigation (e.g. local model re-identification and disk margin analysis).

Quantifying basins of attraction. A general perspective on safety that is compatible with nonlinear systems is the notion of the 'backward reachable set' (BRS) (7, 8). These sets are typically constructed with respect to some (desired) target set of states and, if we take the equilibrium (i.e. $x = \dot{x} = 0$) as such a target set, then the BRS describes the initial states from which system trajectories can start at in order to arrive at the equilibrium within an allocated finite time horizon, T. While this is not a direct quantification of system instability, a shrinkage of the BRS can occur as an instability is approached since the system becomes less capable of maintaining the desired equilibrium (i.e. the safe operating region contracts). It is therefore frequently used in the context of system safety to establish a set of safe operating states (9). Intuitively, the larger the BRS, the more resilient the system is as it can recover from more states. From the perspective of CSD, the system has a larger basin of attraction.

Another common approach towards determining the basin of attraction of a nonlinear control system is through Lyapunov stability analysis. Briefly, the goal is to determine whether the trajectories of a controlled system either remain near an equilibrium or approach it asymptotically as time approaches infinity. This relates naturally to the role of the rate of recovery dynamics in CSD: before the bifurcation point, the non-zero rate of recovery ensures that the system's trajectory converges to the equilibrium point at some point in time. However, at the bifurcation point, the rate of recovery vanishes implying that a state trajectory is no longer guaranteed to return to, or even remain near, the equilibrium; the system is no longer stable in the Lyapunov sense. Though Lyapunov stability theory is a useful tool for assessing the time-invariant (asymptotic) stability of a nonlinear control system, we use the BRS here to make explicit the link between CSD and the reachability of the mass spring damper system in a time-varying setting. Moreover, as we are concerned with the changing rate of recovery of a system, we favor approaches that explicitly involve a finite temporal element.

To this end, we compute the BRS for the actuated mass spring damper system, under the different feedback gain values, for a time horizon of 12 seconds (which affords enough time for the system to reach its steady states for the given system constants in SI Text: Actuated mass spring damper). The resultant BRS are depicted in fig. S5 **c** for which a clear shrinkage in the BRS can be observed as the gain, K, is increased towards instability. This shrinkage mirrors that of the disk margin and the trends in the generic indicators of CSD (i.e. AC1). Indeed, CSD is known to reflect a change in a system's basin of attraction (10, 11) and is therefore a rather intuitive, yet generic, proxy of the BRS.

To further illustrate this connection between the BRS and CSD, we compute the BRS as a function of the critical parameter for the actuated mass spring damper system and a nonlinear ecological system. Specifically, we employ the resource exploitation model (eq. 14) of Dakos et al. (5) to simulate an ecological system with two alternative stable states. Figure S7 depicts the resultant BRS of the engineered and ecological systems as a function of their critical parameters for various time horizons. The contracting basins of attraction as the respective critical transitions are approached are clearly visible. How the BRS are computed for these systems, alongside the parameters of the over-grazing model, are summarized in SI Text: Control theory for ecological models.

Critical slowing down and control theory. These results demonstrate that critical slowing down relates well to classical notions of robustness in control systems. All these frameworks provide different perspectives on system resilience. While the disk margin and backward reachable set can be used to respectively *quantify* the tolerable perturbations and basin of attraction of a system, they rely on substantial and accurate knowledge of the system model in order accomplish this. Instead, critical slowing down allows us to monitor such changes system characteristics in a generic (i.e. model-free) fashion, at the cost of a lack of a quantification of the exact bounds of attraction or perturbation scale.

Actuated mass spring damper.

System dynamics. Combining eqs. 6 and 7, the mass spring damper system with actuator dynamics can be written in state space form as:

$$\begin{bmatrix} \dot{x} \\ \ddot{x} \\ \dot{u} \end{bmatrix} = \begin{bmatrix} 0 & 1 & 0 \\ \frac{-k}{m} & \frac{-d}{m} & \frac{1}{m} \\ 0 & 0 & -\frac{1}{\tau} \end{bmatrix} \begin{bmatrix} x \\ \dot{x} \\ u \end{bmatrix} + \begin{bmatrix} 0 \\ 0 \\ \frac{1}{\tau} \end{bmatrix} \widetilde{u} \qquad [9]$$

We elect the parameters $k = 0.8, d = 0.9, m = 1$ and $\tau = 5$. Under the control law in eq. 8, the mass spring damper system is stable for $K < 4.59$, when K is purely positive[*].

Simulations. We collect synthetic measurement data of the controlled system via stochastic simulations using GRIND[†] for MATLAB (2024b). We express this as a system of equations involving the controller (eq. 8) and system (eq. 9). In this configuration, the controller can be seen as an 'internal' self-regulation mechanism of the dynamical system. This controlled system is subject to stochastic forcing via Brownian motion where noise enters the system additively. Additive noise is chosen here to reflect the presence of non-zero (sensor) noise at the trivial equilibrium states (i.e. $x = \dot{x} = 0$), which the controller is seeking to drive the system towards. This produces persistent perturbations, even at equilibrium, that the controller endeavors to reject. Such noisy measurements are prevalent across many engineered systems (2, 3).

A batch of 100 simulations are run for each discrete gain value, $K \in \{0.5, 1.0, 1.5, 3.0\}$, on approach to instability ($K > 4.59$). All other system parameters are kept constant. Additive noise of magnitude $\sigma = 0.25$, affecting only $\dot{x}$ and $\ddot{x}$, is injected into

[*] Note that, the system is also unstable for $K < -0.8$. However, $K < 0$ switches the controller behavior from regulation (negative feedback, which drives state to zero) into amplification (positive feedback). As we are concerned with the regulation task, we assume $K > 0$ for a valid control law.

[†] Freely available here: https://sparcs-center.org/grind/

the state equation (eq. 9) as a Wiener process. Each simulation is initialized with a unique random number generator seed and runs for 50 seconds with a time step of 0.1 seconds. For each simulation, the system states (i.e. position, x, and velocity, $\dot{x}$) are recorded at every time step to compose the synthetic measurements.

Indicators of instability.

Disk margin. We use the disk margin to determine the stability margins of the controlled mass spring damper system. Given a (linear) system model, $H(s)$, and a controller, $K(s)$, expressed in the Laplace domain, the sensitivity function[‡] $S(s)$ of the system may be obtained through eq. 10.

$$S(s) \;=\; \frac{1}{1 \;+\; H(s)K(s)} \qquad [10]$$

Subsequently, the disk margin – which accounts for simultaneous changes to the gain and phase margins of the system – can be obtained through eq. 11 for single-input-single-output systems (as is the case for our example mass spring damper system). Seiler et al. (6) provide a detailed tutorial on how to calculate the disk margin, including systems with more inputs and outputs.

$$DM \;=\; \frac{1}{||S - \frac{1}{2}||_\infty} \qquad [11]$$

Backward reachable set. Though the backward reachable set is invaluable in theory, computing such sets in practice presents a challenge due to the dependence on an accurate system model and computational intractability (when using standard level-set methods) for high-dimensional systems (9). Nonetheless, we may leverage the closed-form solution of linear autonomous systems (eq. 12) to easily compute the backward reachable set of eq. 9. We use the state transition matrix, $\widetilde{A}$, of the controlled mass spring damper system (eq. 13) to determine the evolution of state trajectories starting from $x\left(t_0\right)$ that terminate after $\left(T - t_0\right)$ seconds at $x\left(T\right)$.

$$x\left(T\right) = e^{\widetilde{A}\left(T - t_0\right)} x\left(t_0\right) \qquad [12]$$

$$\widetilde{A} = \begin{bmatrix} 0 & 1 & 0 \\ \frac{-k}{m} & \frac{-d}{m} & \frac{1}{m} \\ -K\frac{1}{\tau} & 0 & -\frac{1}{\tau} \end{bmatrix} \qquad [13]$$

The backward reachable set is constructed as the set of initial system states (i.e. position, x, and velocity, $\dot{x}$) which terminate at the equilibrium point (i.e. $x = \dot{x} = 0$) by the end of the allotted time horizon. In fig. S5 **c**, we initialize the system states on a grid and choose $T = 12$ seconds, affording enough time for the system to reach its steady state values. Moreover, the effects of disturbances and noise on the backward reachable set is ignored here since we aim only to give an impression on how the backward reachable set changes as the system progresses towards instability, rather than a robust quantification of its exact bounds.

Critical slowing down. As with the quadrotor, we rely on the lag-1 autocorrelation (AC1) metric of critical slowing down (CSD) as our generic indicator of instability. Moreover, we compute these indicators for both the position, x, and velocity, $\dot{x}$, of the mass spring damper system in order to explicitly show the relation between CSD and the changes in the backward reachable set of the system. Though we incrementally shift the mass spring damper towards instability, we nonetheless compute the generic indicators over moving windows sliding along the (synthetic) measurement data. The result is two continuous indicators of instability, one for the position and the other for velocity. Such continuous monitors are appropriate for prompt instability detection in real-world monitoring applications where changes to the system dynamics can occur during operation or between sets of operations (e.g. due to changing environmental conditions).

To this end, a moving average detrender is used to remove trends from both state variables individually along a moving window sliding across their respective timeseries. An averaging window of 5 samples (equal to 0.5 seconds) results in suitable detrending (i.e. produces an autoregressive process). Subsequently, the AC1 is calculated over a rolling window of 157 samples (equal to 15.7 seconds) running along each detrended timeseries individually. This window size is chosen based on the slowest eigenvalues of the uncontrolled system (i.e. eq. 9 with $\widetilde{u} = 0$) such that their dynamics can be observed within the observation window. Within this window, the AC1 coefficient is estimated through a Pearson correlation of the detrended signal with itself lagged by one sample.

Finally, the AC1 values of each simulation at a fixed feedback gain, K, are concatenated into a single representative AC1 histogram of all the values observed under K. These histograms are depicted in fig. S5 **a**. The entire AC1 construction process, from measurement to AC1 histogram, is illustrated in fig. S6 **a**.

[‡] The sensitivity function is often used to study how variations in the system dynamics affect the controlled system as a whole (i.e. combination of controller and system dynamics).

Control theory for ecological models.

Vegetation over-grazing model. As an example of an ecological system, we consider the resource exploitation model (eq. 14) which exhibits critical slowing down (CSD) (5, 12). This model is given by:

$$dV = \left(rV \left(1 - \frac{V}{K}\right) - c\frac{V^2}{V^2 + h^2} \right) dt + \sigma V dW \qquad [14]$$

where the vegetation biomass, V, grows logistically with growth rate, r, up to a carrying capacity, K. The consumption rate of the vegetation is given by c with h denoting the half-saturation constant. This system is subject to multiplicative white noise, dW, with intensity $(\sigma V)^2$ per time step, dt. Following (5) we adopt $r = 1, h = 1, K = 10$ and $\sigma = 0.03$.

Though it is well-established that this ecological system experiences CSD (5, 10), we nonetheless calculate the generic indicators for this system to highlight the consistency in the behavior of these indicators across both ecological and controlled systems. Here, we construct a synthetic ecological data set through a series of stochastic simulations of eq. 14 for different consumption rates ($c = \{1.00,\ 1.50,\ 1.75,\ 2.00\}$) held fixed throughout a simulation. For each value of c, 50 independent simulations are run using GRIND for MATLAB (R2024b), each with a unique pseudo-random number generator seed. Each simulation runs for 1500 days with a time step of $dt = 1$ days.

To mirror the processing steps of the controlled systems studied here, we remove slow trends from the vegetation biomass measurements through a moving average detrender. An averaging window size of 40 days produces a suitable first order autoregressive process. Using this detrended timeseries, the lag-1 autocorrelation (AC1) metric of CSD is calculated over a sliding window of 500 days. Within this 500 day window, the AC1 coefficient is estimated through a Pearson correlation of the detrended vegetation biomass with itself lagged by one sample. This process is illustrated in fig. S6 **b**.

Backward reachability in ecology. As the bifurcation points of eq. 14 are approached, the basin of attraction of the associated equilibrium points shrinks. This is reflected by a shrinkage in the backward reachable set (BRS) en route to a bifurcation, shown in fig. S7 **a**. Also depicted, for reference, is the contracting BRS of the actuated mass spring damper system (eq. 8 and eq. 9) as it shifts towards instability. For both systems, the BRS in fig. S7 is computed using a system model evaluated on a grid of the system states and critical parameter. For each critical parameter value, the system is initialized, without noise, over a mesh of the states and is simulated forward for the different time horizons. These deterministic simulations are run in Python (3.13). An initial state qualifies for the BRS if its terminal state is at the equilibrium states by the end of the time horizon (i.e. simulation).

Though the change in morphology of the BRS due to variations in the critical parameter can be non-trivial, such as the 'ghost' (white) regions in fig. S7 **a**, the shrinkage of the set alone can be indicative of an approaching critical transition. To this end, such reachable sets may be used alongside a time horizon to help design (conservation) policies.

When afforded the luxury of representative models of an (ecological) system, the BRS can give temporal insights into policy design. For example, how long before an ecological system can be expected to return to equilibrium and, if this time frame is too long, what 'controllers' (i.e. conservation policies) can be followed to expedite this recovery. Contrarily, if time is not a concern, what strategies are least intrusive, most resilient, or most convenient to implement? This can help motivate the trade-off between different conservation policies. In a similar capacity, the BRS can be used to determine viable, perhaps even counter-intuitive, paths to recovery for multi-state systems through the gradients afforded by the BRS.

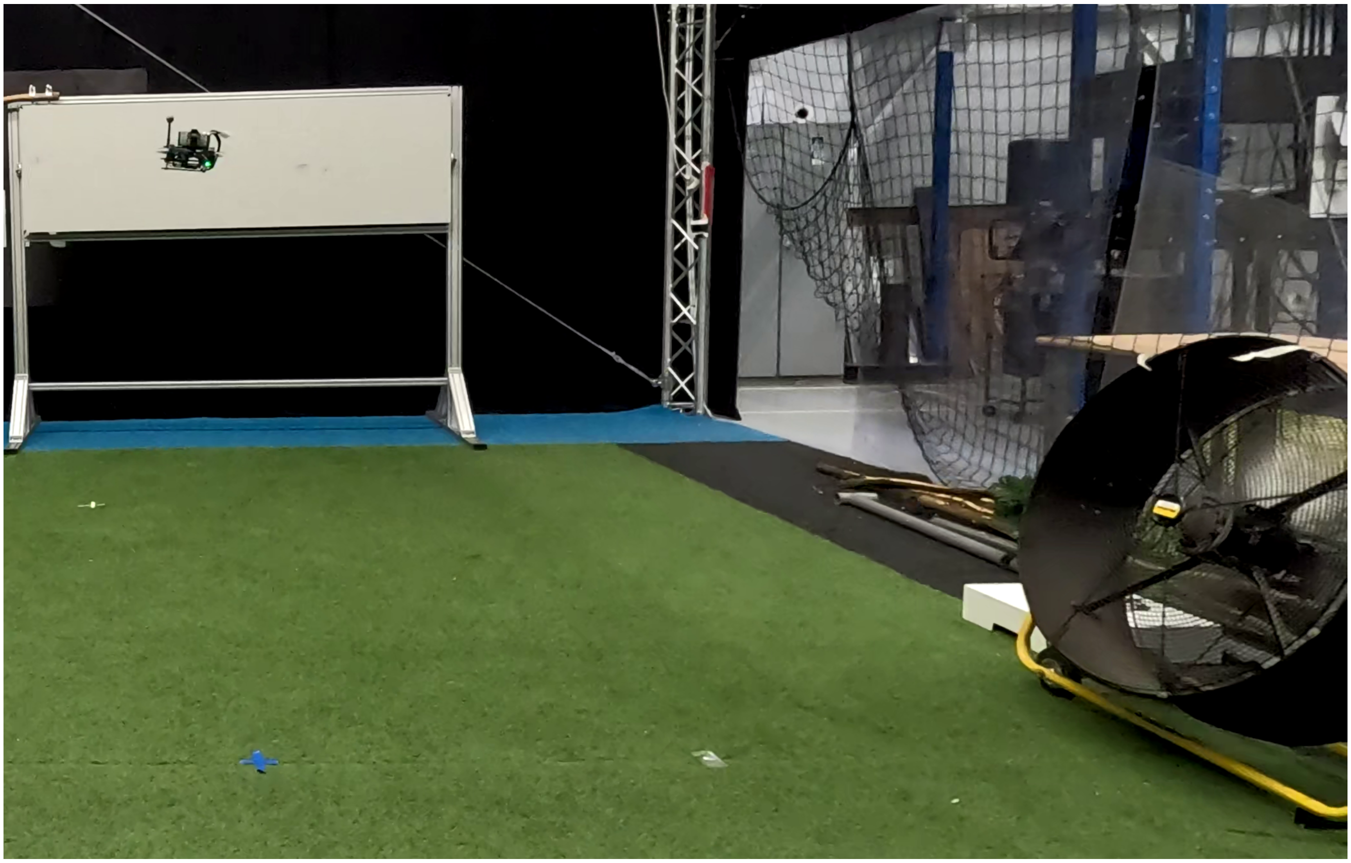

Fig. S1. Windy flight experiments. Snapshot of one of the autonomous flight experiments with wind generated by a large fan.

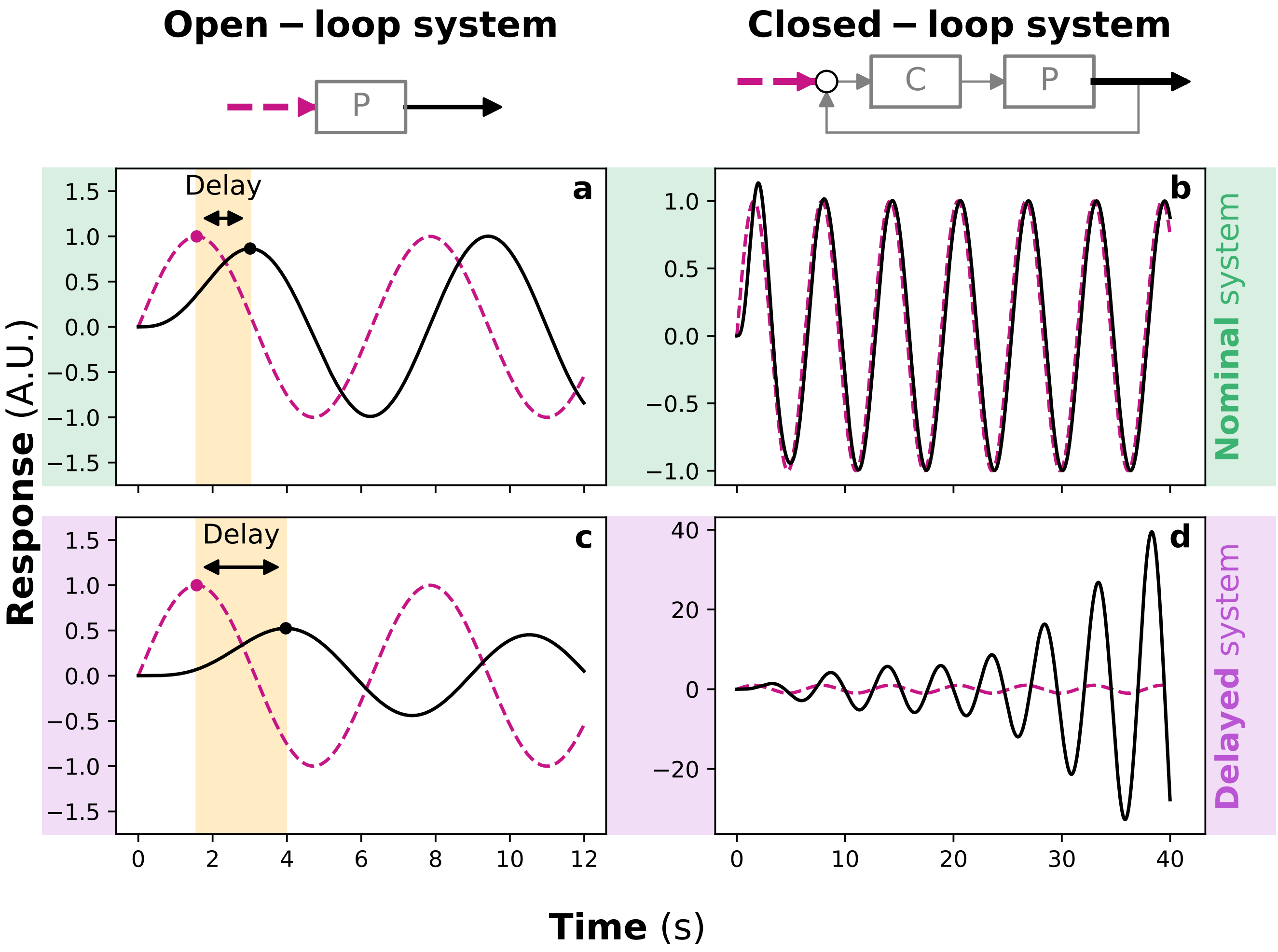

Fig. S2. Effect of delay in controlled systems.

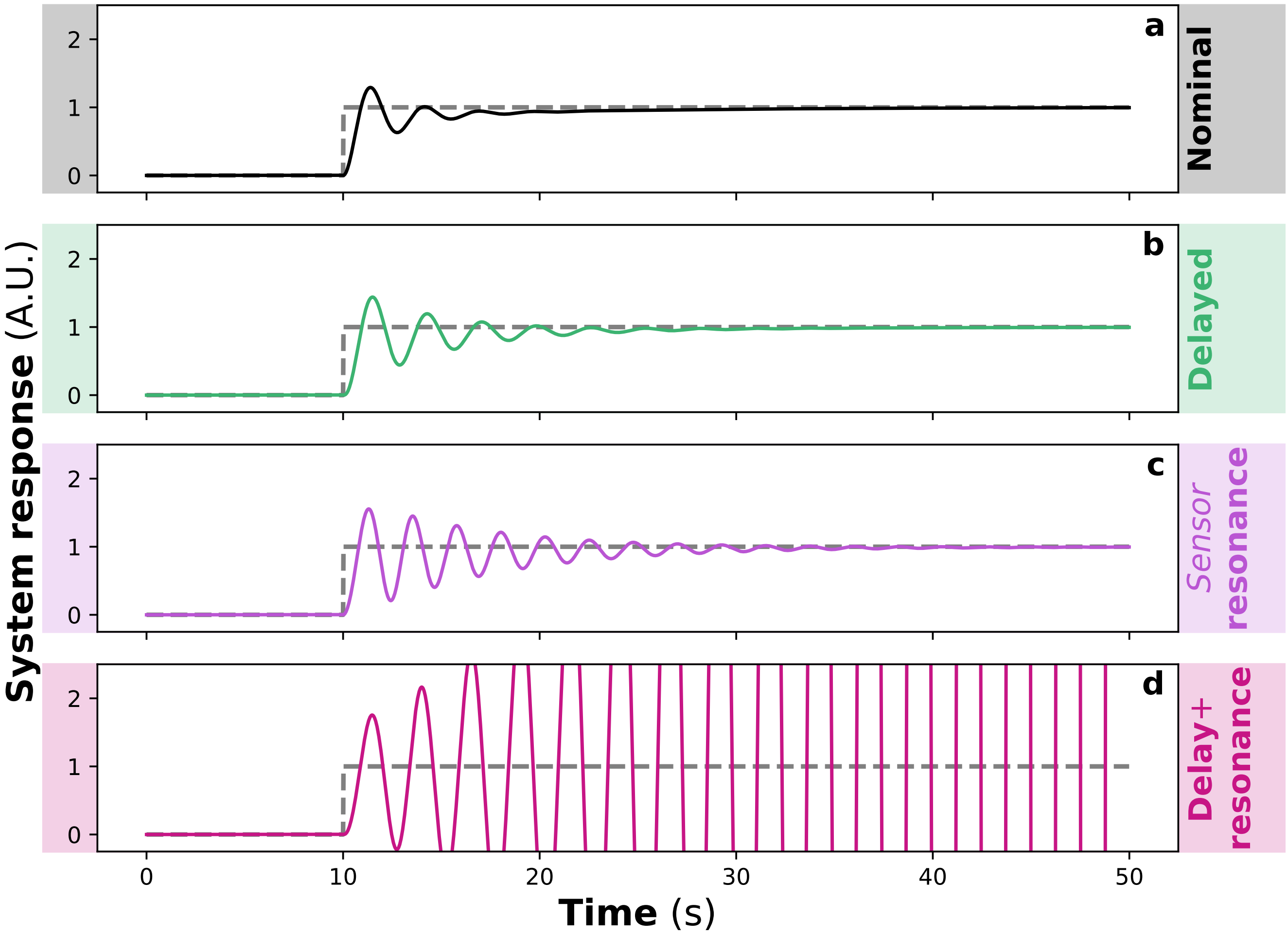

Fig. S3. Effect of state measurement imperfections on controller behavior.

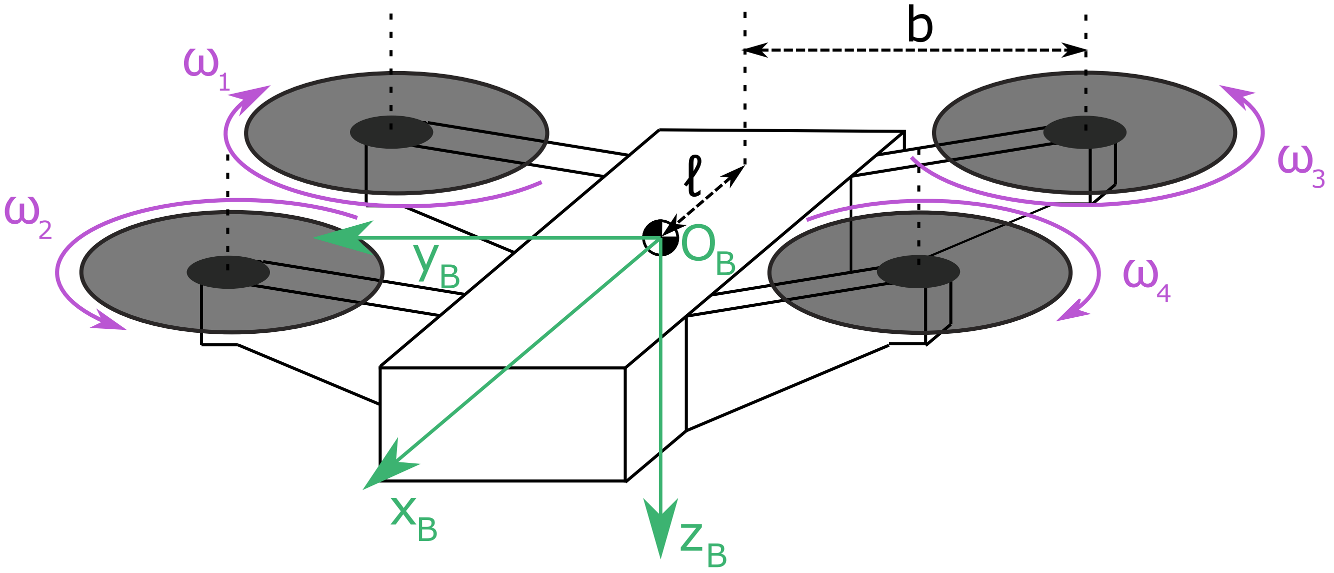

Fig. S4. Coordinate frame and geometric properties of the quadrotor. The rotor layout utilized by both our quadrotors is indicated by $\omega \in \{\omega_1, \omega_2, \omega_3, \omega_4\}$ in purple with rotor 1 (ω_1) spinning clockwise. The forward-right-down body reference frame is shown in $O_B = \{x_B, y_B, z_B\}$. b and ℓ describe the x and y moment arms, respectively.

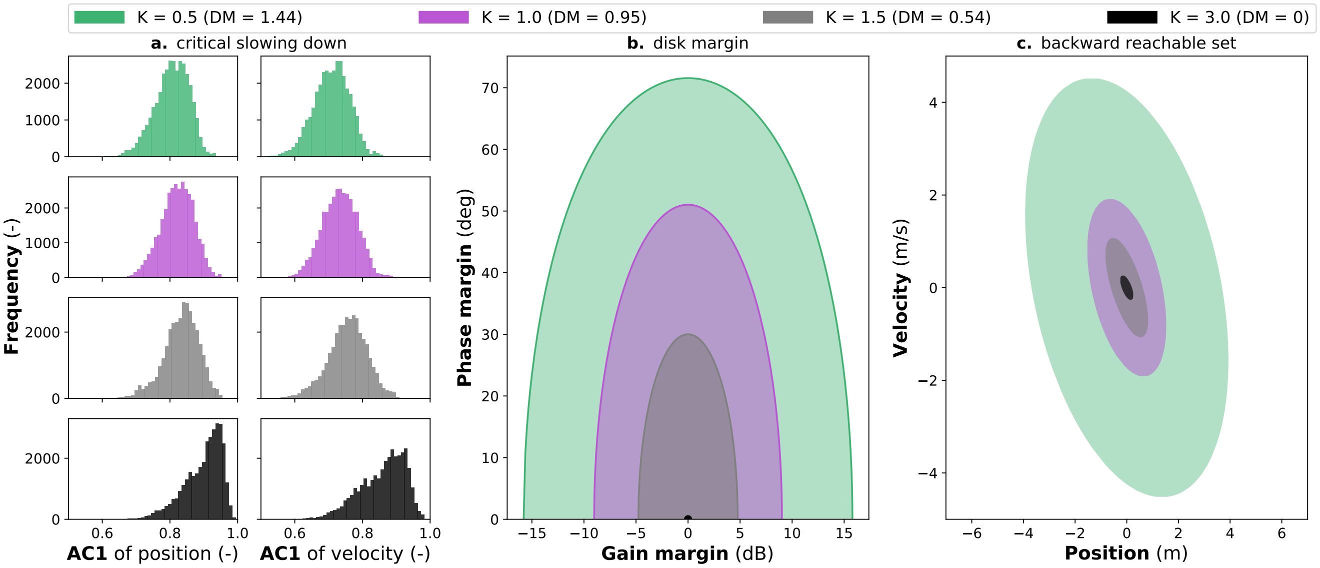

Fig. S5. Indicators of controlled system resilience. An actuated (linear) mass spring damper system is used to compare various indicators of controlled system resilience subject to different control gains, K, which govern the stability of the system. **a** depicts the critical slowing down lag-1 autocorrelation (AC1) metric, which shows the characteristic approach to one for both state variables as the instability is approached incrementally. **b** and **c** are classical control theoretic measures of system robustness and denote the disk margin (DM) and backward reachable set (with an arbitrary time horizon of 12 seconds) respectively. Both exhibit a shrinkage in area as the instability is approached, indicating a loss of robustness (i.e. resilience).

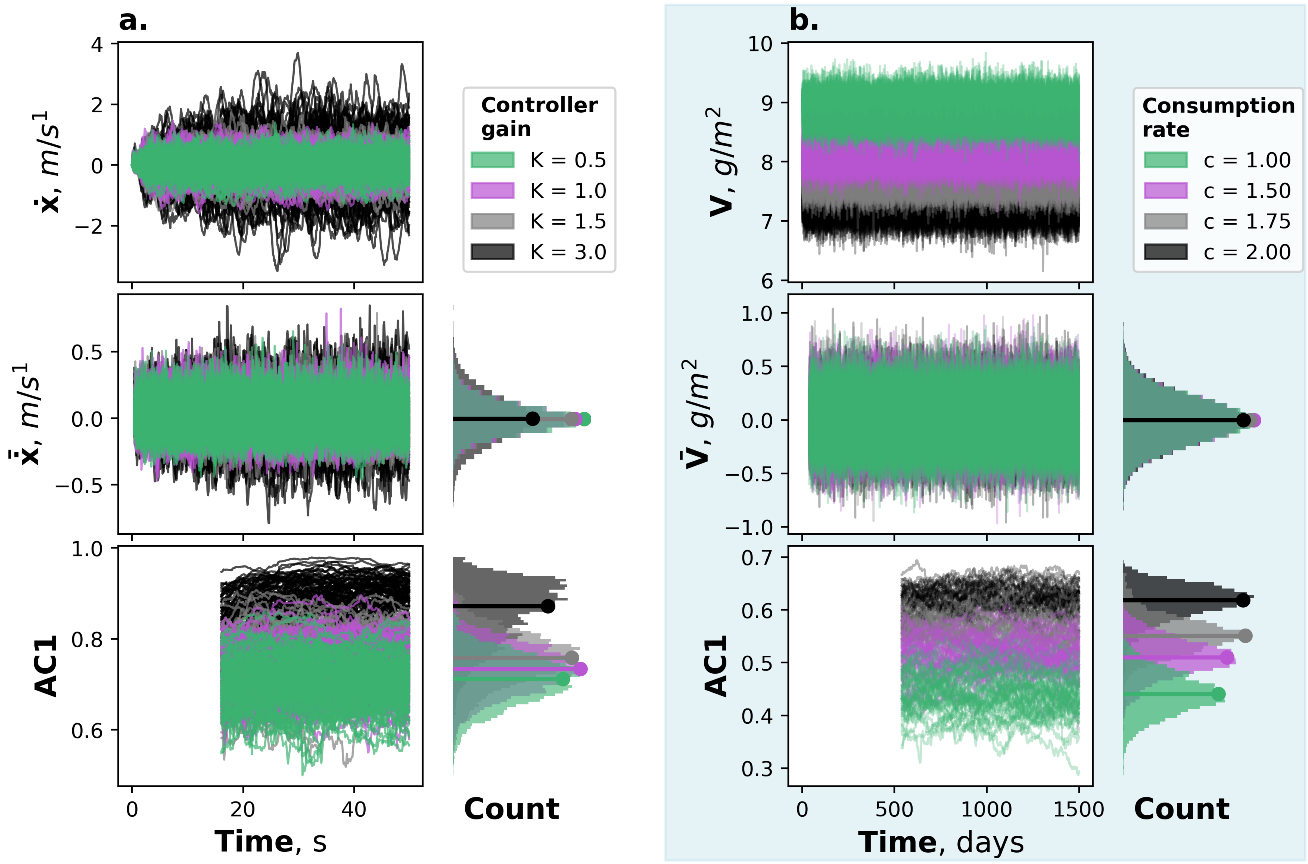

Fig. S6. Generic indicators of sudden changes in dynamical system behavior. Illustrated are the generic indicator construction steps from a state measurement to the lag-1 autocorrelation (AC1) metric of critical slowing down for the controlled mass spring damper system (**a**) and resource exploitation ecological system (**b**). Synthetic measurement data is collected through multiple simulations using GRIND for MATLAB with white noise perturbations. The first row of plots show the raw measurement signals as a function of the critical parameter values on approach to a sudden shift in dynamical system behavior. Slow trends in these signals are removed via a moving average filter that slides along the measurement data to produce a valid first order autoregressive process. Subsequently, the AC1 is estimated via the Pearson correlation coefficient taken within yet another sliding window over the detrended timeseries. The resultant AC1 histograms depict the characteristic increase in AC1 as the tipping point is approached (from green, to purple, to grey, to black).

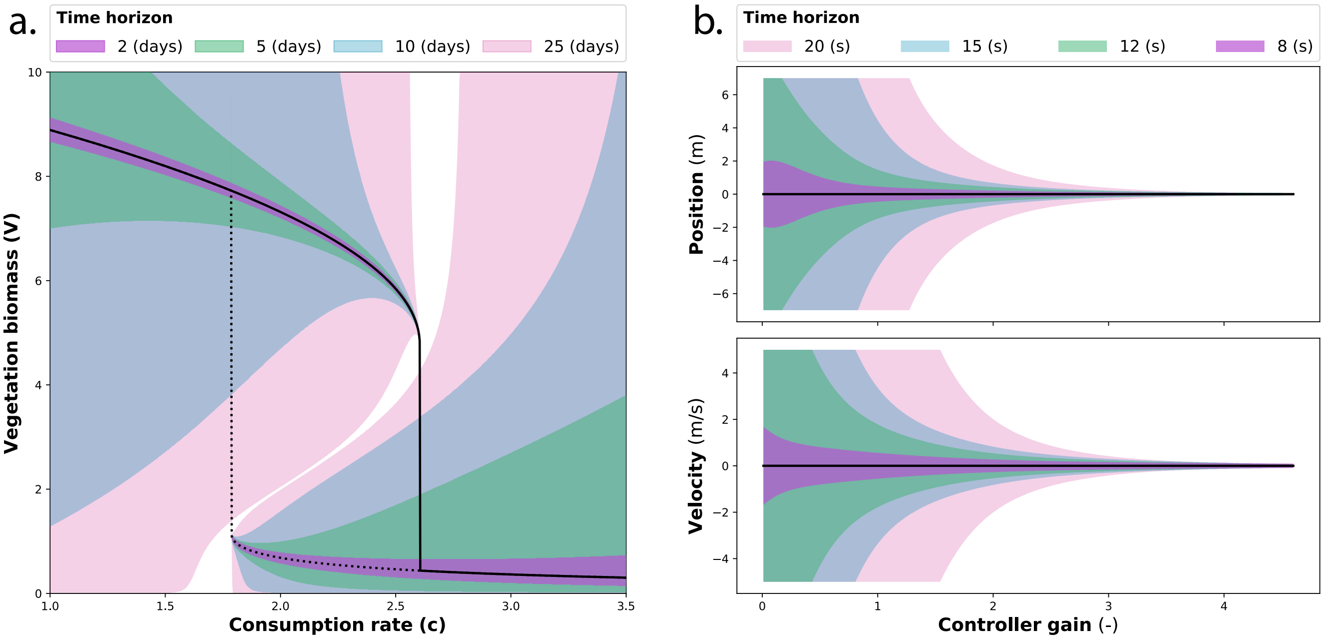

Fig. S7. Change in backward reachable set as a function of the critical parameter. The backward reachable set (BRS) is shown for the resource exploitation ecological system (eq. 14) in **a**. For reference, the BRS of a controlled mass spring damper system (eq. 8 and eq. 9) is also shown in **b**. For both systems, a clear contraction in the BRS is observed as the transition points are approached, for all time horizons. The BRS of these systems are computed through deterministic simulations of the system along a grid of critical parameter values. For each critical parameter value, the system states are initialized on a grid, without noise, and are simulated forward in time for the different time horizons. The BRS is composed of the initial states which terminate at the equilibrium states.

Table S1. Autonomous flight conditions. Overview of autonomous (indiflight) flight tasks of the DragonFly.

Damage location	Damage level	Flight task	Windy	Number of flights
N/A	0%	Hover	No	10
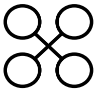	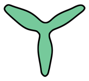	Circle at 1.5m/s	No	10
		Circle at 1.5m/s	Yes	5
		Circle at 3.0m/s	No	5
		Rectangle V1	No	10
		Rectangle V1	Yes	5
		Rectangle V2	No	5
Front right	10%	Hover	No	10
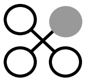	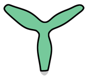	Circle at 1.5m/s	No	5
		Circle at 1.5m/s	Yes	5
		Circle at 3.0m/s	No	5
		Rectangle V1	No	5
		Rectangle V2	No	5
Front right	15%	Hover	No	10
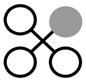	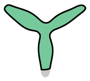	Circle at 1.5m/s	No	5
		Circle at 1.5m/s	Yes	5
		Circle at 3.0m/s	No	4
		Rectangle V1	No	5
Aft right	10%	Hover	No	10
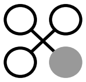	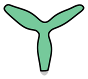	Circle at 1.5m/s	No	5
		Circle at 1.5m/s	Yes	5
		Circle at 3.0m/s	No	5
		Rectangle V1	No	5
Aft right	15%	Hover	No	10
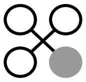	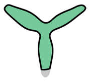	Circle at 1.5m/s	No	5
		Circle at 1.5m/s	Yes	5
		Circle at 3.0m/s	No	3
		Rectangle V1	No	5
Front left	10%	Hover	No	5
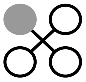	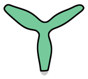	Circle at 1.5m/s	No	5
		Rectangle V1	No	5
		Rectangle V1	Yes	5
Front left	15%	Hover	No	5
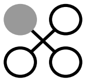	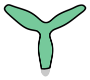	Circle at 1.5m/s	No	5
		Rectangle V1	No	5
		Rectangle V1	Yes	5
Aft left	10%	Hover	No	5
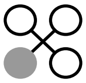	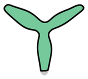	Circle at 1.5m/s	No	5
		Rectangle V1	No	5
		Rectangle V1	Yes	5
Aft left	15%	Hover	No	5
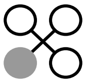	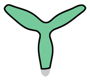	Rectangle V1	No	5
		Rectangle V1	Yes	5

Table S2. Quadrotor properties. Components and physical characteristics of the two quadrotors used in the flight experiments.

	DragonFly	HoverFly
**Drone frame**	Ethix CineRat	Ethix CineRat
**Mass (incl. batteries),** g	494.13	282.56
**Diagonal hub-to-hub diameter,** mm	153	153
**Propeller diameter,** mm	76	76
**Motor**	Emax Eco 1407 2800 kV	Ethix Flat Rat-V2 1507 2800 kV
**Batteries**	Tattu FunFly 22.2V 1300mAh	Tattu R-Line 14.8V 550mAh
**Flight Controller (FC)**	MATEKSYS H743-SLIM V3	MATEKSYS H743-SLIM V3
**FC Software**	INDIFlight	Betaflight 4.3.2

Movie S1. Comparison of flight trajectories of the INDIFlight controlled DragonFly quadrotor at the 0% (white), 10% (green), and 15% (purple) damage levels where loss of control occurs at the 15% damage level (front right rotor).

Movie S2. Shows two perspectives of the inability of the INDIFlight controlled DragonFly quadrotor to maintain stable flight at the 30% damage level. Here we attempt to pilot the drone in manual mode (i.e. piloted flight; removing the dependency on the motion capture system). Nonetheless, the rotor speeds are unstable due to excessive vibrations. This can be heard in the video.

Movie S3. Persistent loss of control of the INDIFlight controlled DragonFly quadrotor with the 15% damage to the aft left rotor. The quadrotor is tasked with following vertices of a rectangle.

Movie S4. Aggressive maneuver during trajectory initialization sequence for circular flights. These cause brief periods of 'near instability' which remain unproblematic for the No and 10% damaged scenarios but lead to loss of stability during the maneuver for the 15% damage scenario.

References

1. M Idrissi, M Salami, F Annaz, A review of quadrotor unmanned aerial vehicles: applications, architectural design and control algorithms. *J. Intell. & Robotic Syst.* **104**, 22 (2022).
2. D Capriglione, et al., Experimental analysis of filtering algorithms for imu-based applications under vibrations. *IEEE Transactions on Instrumentation Meas.* **70**, 1–10 (2021).
3. J Wu, et al., Resonance interference research of mems inertial sensors and algorithm elimination. *IEEE Sensors J.* **22**, 10428–10436 (2022).
4. JD Crawford, Introduction to bifurcation theory. *Rev. Mod. Phys.* **63**, 991–1037 (1991).
5. V Dakos, et al., Methods for detecting early warnings of critical transitions in time series illustrated using simulated ecological data. *PLOS ONE* **7**, 1–20 (2012).
6. P Seiler, A Packard, P Gahinet, An introduction to disk margins [lecture notes]. *IEEE Control. Syst. Mag.* **40**, 78–95 (2020).
7. J Lygeros, On reachability and minimum cost optimal control. *Automatica* **40**, 917–927 (2004).
8. IM Mitchell, AM Bayen, CJ Tomlin, A time-dependent hamilton-jacobi formulation of reachable sets for continuous dynamic games. *IEEE Transactions on automatic control* **50**, 947–957 (2005).
9. S Bansal, M Chen, S Herbert, C Tomlin, Hamilton-jacobi reachability: A brief overview and recent advances. *Proc. IEEE Conf. on Decis. Control. (CDC)* (2017).
10. M Scheffer, et al., Early-warning signals for critical transitions. *Nat. 2009 461:7260* **461**, 53–59 (2009).
11. V Dakos, J Bascompte, Critical slowing down as early warning for the onset of collapse in mutualistic communities. *Proc. Natl. Acad. Sci. United States Am.* **111**, 17546–17551 (2014).
12. K D'Souza, BI Epureanu, M Pascual, Forecasting bifurcations from large perturbation recoveries in feedback ecosystems. *PLOS ONE* **10**, e0137779 (2015).
